# Beneficial metabolic transformations and prebiotic potential of hemp bran and its alcalase hydrolysate, after colonic fermentation in a gut model

**DOI:** 10.1038/s41598-023-27726-w

**Published:** 2023-01-27

**Authors:** Lorenzo Nissen, Flavia Casciano, Elena Babini, Andrea Gianotti

**Affiliations:** 1 Department of Agricultural and Food Sciences (DISTAL), Alma Mater Studiorum - University of Bologna, Piazza Goidanich 60, 47521 Cesena, FC Italy; 2 Interdepartmental Centre of Agri-Food Industrial Research (CIRI), Alma Mater Studiorum - University of Bologna, P.Za G. Goidanich 60, 47521 Cesena, FC Italy

**Keywords:** Microbial ecology, Metabolomics, Biotechnology, Food microbiology

## Abstract

Hemp seed bran (HB) is an industrial food byproduct that is generally discarded. Knowledge on the functional capabilities of HB is limited and it is not known the impact of HB on human colon microbiota, where vegetable fibers are metabolized. In this work, we investigated in depth the prebiotic potential of HB and HB protein extract hydrolyzed by alcalase (HBPA) in comparison to fructooligosaccharides (FOS) after human distal colonic fermentation using MICODE (multi-unit in vitro colon gut model). During the 24 h of fermentation, metabolomics (SPME GC/MS) and microbiomics (MiSeq and qPCR) analyses were performed. The results indicated that HBPA on a colonic fermentation had a higher prebiotic index than HB (*p* < 0.05), and slightly lower to that of FOS (*p* > 0.05). This feature was described and explained as HBPA colonic fermentation produces beneficial organic fatty acids (*e.g.* Pentanoic and Hexanoic acids); reduces detrimental phenol derivates (*e.g. p*-Cresol); produces bioactives VOCs (*e.g.* Acetophenone or 4-Terpineol); increases beneficial bacteria (*e.g.* 1.76 fold and 2.07 fold more of *Bifidobacterium bifidum* and *Bacteroides fragilis*, respectively) and limits opportunistic bacteria (*e.g.* 3.04 fold and 2.07 fold less of *Bilophila wadsworthia* and *Desulfovibrio*, respectively). Our study evidenced the prebiotic role of HB and HBPA, and within the principles of OneHealth it valorizes a byproduct from the queen plant of sustainable crops as a food supplement.

## Introduction

Hemp seed is a powerhouse of nutrients and a mine of bioactives, bearing some exceptional issues as: being sugarless, low in glycemic, gluten free, rich in balanced ratio of PUFA, neuro strengthener, cardiovascular protection, etc^1^. A principal criterium of OneHealth philosophy is sustainability of the food industry, that should be guided to the reduction of wastes and by the exploitation of byproducts. Considering this scenario, we recently have set attention on Hemp seed bran (HB), which is an unexplored byproduct of the industrial processing of hemp seed. A better exploitation of the industrial food chain production is a need, and the valorization of HB, which is treated as a byproduct and mainly discarded, is our main focus. Indeed, we previously characterized HB for its ability to foster the growth of beneficial bacteria and to exhibit potent prebiotic potential depending on its level of processing^2,3^. New functional ingredients for bakery foods, thereby increasing their nutritional value were also obtained^3^.

Considering the use of HB as an innovative and low-cost source to formulate healthier and value-added foods, its impact on human colon microbiota and an exhaustive explanation and comprehension of its prebiotic potential are situations to investigate on. In fact, the latest definition of prebiotics^4^ indicates that there are other compounds than oligosaccharides that can get the claim, such as polyphenols and terpenes, of which HB is rich^3^. By definition, prebiotics are degraded by colon microbes and influence the whole microbiota; directly feeding the commensals group and fostering probiotics towards eubiosis and consequently to host health^4^. The action of a prebiotic is also directed to the limitation of opportunistic bacterial groups that produce toxic catabolites, such as phenols and some indoles^5^.

In this state, HB and its enzymatic hydrolysates could play an important role, because, as we have previously demonstrated in in vitro studies with type strains bacteria, other than carbohydrate compounds, HB brings many bioactives, *e.g. p*-Cymene, Caryophillene, 4-Terpineol, Acetophenone, Myrtenal^3^.

In vitro gut models are considered a proper solution to study the impact of dietary beneficial compounds on human gut microbiota^6,7^. Throughout the assessment of microbiomics (ecological diversities and the shift of the microbiota), metabolomics (volatilome profiling), and inter-omics (correlations of the previous two) it is possible to unveil the cause and effects of the fiber’s functionalities. In this context, in vitro colon fermentation was simulated with the aid of a human colon model, namely MICODE (Multi-unit in vitro Colon Model) a versatile colon model^8,9^ to specifically study prebiotic potential of HB and of alcalase-treated HB protein isolate (HBPA), by simulating the distal part of the human large intestine. HBPA was previously characterized for its antioxidant and antiypertensive properties^2^, also related to the presence of bioactive peptides originated from protein hydrolysis^10^.

We used MICODE with fecal samples from three healthy donors for a short-term colonic fermentation protocol (24 h) of HB and HBPA in comparison to prebiotics (FOS) as positive control and to a blank control. Aiming at the understanding of the potential health benefits of HB alcalase hydrolysates, an inter-omic approach coupling microbial genomics (qPCR and Illumina sequencing) and metabolomics (SPME GC–MS) was adopted, focusing on ecological indicators such as: (i) microbial biodiversity, (ii) microbial eubiosis, (iii) prebiotic index, (iv) production of prebiotic compounds, such as SCFAs and MCFAs, (v) reduction of detrimental compounds, such as phenols and p-Cresol, (vi) presence of bioactive volatiles, such as Borneol and Acetophenone, and (vii) shift in those bacterial taxa specialized in fiber degradation or in proteolytic fermentations.

## Results and discussion

### Quality controls for the validation of MICODE protocol

To validate the MICODE experimental approach in the version of fecal batch of the human proximal colon, we chose to monitor and check some parameters as quality controls (QC) related to metabolites and microbes at the end of fermentations, and in comparison, to the baseline. QCs adopted were; (i) the *Firmicutes/Bacteroidetes* ratio (F/B), which is related to health and disease^11^, was maintained at a low level, confirming the capacity to simulate a healthy in vivo condition for 24 h. (ii) The presence of *Archea* (*e.g*., *Methanobrevibacter smithii* and *Methanosphaera stadtmanae*), which are pretty sensible to oxygen content^12^, was retained from the baseline to the end point in each vessel and repetition, indicating that the environmental conditions were strictly maintained. (iii) Good’s rarity index of alpha biodiversity remained similar during time of fermentation (*p* > 0.05), indicating enough support to the growth of rare species. (iv) Observed OTUs richness index scored approximately 400 OTUs at the end point. (v) The paradigm of prebiotics was confirmed when the positive control (FOS) was applied on MICODE; high probiotic and SCFAs increases and limitation of enteropathogens. (vi) Each GC/MS analysis had quantified some stool-related compounds (urea, 1-propanol, and butylated hydroxy toluene), that ranged across the complete chromatogram and were adsorbed at the same retention times.

### Changes in bacterial alpha and beta diversities

The microbiota diversity indices were analyzed to study the impact of HPBA on microbial population, to assess population’s stability during fermentation, and to compare its microbiota to that of other bioreactors (Figure S1). The baseline of value was compared to the endpoints of fermentation of different treatments. It is undisputable that a part of the effect of reduction in richness (Observed OTUs) was derived by the passage from in vivo to in vitro condition, but the focus must be set on the different trend that other alpha diversity indices had. For example, abundance (Chao 1) for HBPA was significantly higher at the end of fermentation (*p* < 0.05), while a not significant reduction was seen for HB or FOS. Surges in evenness (Shannon) were seen for HB (*p* > 0.05) and HPBA (*p* < 0.05), but no changes were seen in dominance (Simpson) (*p* > 0.05), while oppositely, FOS decreased in evenness (*p* > 0.05) and raised in dominance (*p* < 0.05). This output indicates a different performance of HPBA or minorly HB in respect to FOS and is well explained by the trend of dominance that tells that for FOS some taxa overcame others, reducing the uniform distribution of bacterial groups in the microbiota. This effect was already observed and could be justified by the ability of FOS to foster *Bifidobacteriaceae* and make them dominant over the microbiota^8,13^. HBPA and minorly HB instead had an effect with a wider range of bioactivity on more bacterial targets; that higher biodiversity could be seen as an added value on its prebiotic potential.

When the bacterial diversity between samples (beta diversity) was examined with Bray–Curtis analysis, the fecal samples was set distant to the BL, and the BL distant to the end point cases, as demonstrated by principal coordinate analysis (PCoA) based on an unweighted (qualitative) phylogenetic UniFrac distance matrix. This feature confirms that shifts occurred during the experiments. Additionally, the four cases at the end point were relatively distant one to each other. This feature confirms that different shifts occurred from the BL on. So far, the study of biodiversity indicated the ability to keep an eubiosis conditions by fermentations of both the hempseed bran samples, with generally a higher capacity of HBPA in respect to HB. Considering that HBPA should have a higher availability of shorter fiber chains and more unbound saccharides due to the action of alcalase treatment^2^, that result could indicate that HBPA is generally more appetible for colonic fermentation than HB.

### Changes in taxa abundances at the phylum level

The total sequence reads used in this study were classified into eight phyla and one unassigned (Table S2). In any tested sample, the core microbiota was represented by five taxa: three with a relative abundance higher than 10% (*Firmicutes*, *Bacteroidetes*, and *Actinobacteria*) and two lower than 3% (*Proteobacteria* and *Verrucomicrobia*). Anyhow, just *Firmicutes*, *Bacteroidetes*, and *Proteobacteria* underwent significant changes in comparison to the baseline (*p* < 0.05).

As a general parameter for microbiota eubiosis we chose the famous ratio *Firmicutes*/*Bacteroidetes* (F/B), and we considered the differences from the baseline to the end point. Within this ratio a value over two is usually referred to microbiota dysbiosis^11,14^. The fecal samples at the baseline had a F/B of 1.66 and this eubiosis condition was maintained by HBPA (1.55), by HB (1.62) and FOS (0.73), although significantly just for the latter (*p* > 0.05). These results indicate that during the time of fermentation, HB and HBPA did not perturb the colon core microbiota of healthy donors but was able to provide a substrate that meet the energetic expenditure of the microbiota, keeping an eubiosis condition.

### Changes in taxa abundances at the species level

A dataset of significant OTUs changes relative to the family level is reported in Table S3. Anyhow, we focused the discussion on results obtained at the specie taxonomic level, where 113 OTUs were constructed and assigned to microbial taxa (cutoffs 0.001%). Of these, 113 were identified at the baseline, while 106, 102, 96, and 100 were identified at the endpoint of fermentations with HPBA, HB, FOS, and the blank control, respectively. Then a dataset of 41 microbial OTUs was selected and tested for ANOVA group comparison in respect to the baseline (*p* > 0.05). Among these, 31 variables were significant and their Log_2_ fold changes in respect to the baseline were compared by post-hoc test (Table 1). The 41 OTUs selected were those that recorded shifts after fermentation and that from literature are susceptible to the effect of prebiotic or fiber substrates. We have included even three OTUs of *Archea* relative to QC of the experiments (previously discussed).

**Table 1:** Abundances (% ± S.D.) and changes in phylum taxa (Log_2_ F/C) after 24 h in vitro fecal batch culture fermentations from healthy donors and administrated with HBPA, HB, and FOS as the substrates, and also including a blank control.

Taxon	% Relative abund	Log_2_(F/C) changes at the end points (24 h)				*p* value*
	Baseline mean	HBPA	FOS	HB	BC	
Quality controls						
* Archaea;Other*	0.001 ± 0.001^b^	3.27^a^	2.07^ab^	3.07^a^	n.d	0.03749
* Methanobrevibacter;s__smithii*	0.486 ± 0.411	− 4.27	− 5.88	− 2.27	− 9.10	0.45636
* Methanosphaera;s__stadtmanae*	0.003 ± 0.004	0.05	− 1.14	0.12	− 1.08	0.92717
Prebiotic sensitive (beneficial and commensal taxa)						
* Bifidobacterium;s__adolescentis*	4.414 ± 1.743^b^	0.38^b^	1.08^a^	0.21^b^	− 0.27^b^	0.03883
* Bifidobacterium;s__bifidum*	0.974 ± 0.177^b^	1.76^a^	1.67^a^	1.16^a^	− 0.25^b^	0.00132
* Bifidobacterium;s__longum*	2.744 ± 0.544	0.45	0.55	0.22	− 0.04	0.15503
* Bacteroides;s__acidifaciens*	0.115 ± 0.009^b^	1.07^a^	1.39^a^	0.29^b^	− 0.27^b^	0.00052
* Bacteroides;s__caccae*	0.713 ± 0.086^b^	1.12^a^	1.46^a^	0.22^b^	− 0.79^b^	0.00113
* Bacteroides;s__fragilis*	0.238 ± 0.188	2.07	1.51	1.01	− 1.85	0.05038
* Bacteroides;s__thetaiotaomicron*	0.393 ± 0.093^c^	4.32^a^	2.90^b^	3.30^a^	0.78^c^	0.00001
* Bacteroides;s__uniformis*	3.583 ± 0.301^c^	1.39^b^	2.92^a^	0.39^c^	− 2.84^d^	0.00001
* Parabacteroides;s__distasonis*	0.666 ± 0.270^b^	2.32^a^	2.95^a^	1.84^a^	0.49^b^	0.00109
* Enterococcus;s__durans*	0.400 ± 0.670^b^	3.55^a^	4.36^a^	3.01^a^	− 2.10^b^	0.00305
* Enterococcus;s__faecalis*	0.005 ± 0.007^b^	6.77^a^	− 1.06^b^	4.21^a^	0.81^b^	0.00001
* Lactobacillus;s__casei*	0.015 ± 0.022	1.78	0.77	1.05	0.01	0.35888
* Lactobacillus;s__manihotivorans*	0.021 ± 0.031	1.74	1.30	0.84	0.57	0.37442
* Lactobacillus;s__mucosae*	0.003 ± 0.002^b^	4.48^a^	4.50^a^	3.35^a^	− 0.05^b^	0.00010
* Lactobacillus;s__plantarum*	0.001 ± 0.000^c^	5.00^b^	7.54^a^	4.02^b^	− 0.03^d^	0.00001
* Streptococcus;s__thermophilus*	0.533 ± 0.359	− 0.65	0.43	− 0.45	− 2.06	0.56392
* Roseburia;s__faecis*	0.096 ± 0.035	− 2.00	− 2.61	− 1.80	− 4.55	0.09065
* Faecalibacterium;s__prausnitzii*	1.734 ± 0.770^a^	0.36^a^	0.31^a^	0.12^a^	− 3.22^b^	0.01857
* Akkermansia;s__muciniphila*	0.903 ± 0.122^a^	0.30^a^	0.55^a^	0.19^a^	− 4.82^b^	0.00869
Prebiotic sensitive (opportunistic taxa)						
* Streptococcus;s__pseudopneumoniae*	0.080 ± 0.073	− 5.73	− 4.93	− 5.02	− 0.54	0.51847
* Bilophila;s__wadsworthia*	0.149 ± 0.019^b^	− 3.04^c^	− 2.12^c^	− 2.04^c^	2.67^a^	0.00006
* Citrobacter;s__freundii*	0.051 ± 0.030	− 0.45	− 5.29	− 0.66	1.67	0.05761
* Escherichia;s__albertii*	0.064 ± 0.042^b^	− 0.19^b^	− 0.59^b^	− 0.17^b^	3.39^a^	0.00202
* Desulfovibrio;s__*	0.395 ± 0.117^a^	− 2.07^b^	− 1.59^b^	− 1.66^b^	0.06^a^	0.04574
* Peptostreptococcaceae;g__Clostridium;s__*	0.062 ± 0.053	− 0.41	− 3.24	− 0.56	1.42	0.23301
Vegetal Fiber sensitive (positive)						
* Blautia;s__*	6.422 ± 1.734	− 6.20	− 3.78	− 4.13	− 2.40	0.05643
* Blautia;s__obeum*	0.977 ± 0.205^a^	− 4.88^b^	− 4.15^b^	− 4.10^b^	− 0.81^ab^	0.02860
* Ruminococcus;s__gnavus*	2.203 ± 0.720^a^	− 5.38^b^	− 3.11^b^	− 4.11^b^	− 0.28^a^	0.04652
* Ruminococcus;s__torques*	0.694 ± 0.607	− 7.26	− 9.04	− 6.06	− 0.66	0.47420
* Tepidibacter;s__*	1.886 ± 0.445	− 0.93	− 2.17	− 0.15	0.43	0.06638
* Oscillospira;s__*	2.085 ± 0.119^b^	2.51^a^	− 3.03^c^	0.73^b^	− 1.79^c^	0.00001
* Megasphaera;s__elsdenii*	3.386 ± 2.635	− 4.05	0.43	− 2.23	− 0.86	0.54442
* Collinsella;s__aerofaciens*	2.389 ± 0.747^a^	− 2.35^b^	− 1.90^b^	− 1.44^b^	0.54^a^	0.04331
* Eggerthella;s__lenta*	0.053 ± 0.015^a^	− 1.95^b^	− 3.74^b^	− 1.63^b^	0.39^a^	0.04445
* Coprobacillus;s__cateniformis*	0.042 ± 0.027^b^	4.35^a^	4.66^a^	4.00^a^	− 2.60^b^	0.00006
* Sutterella;s__*	1.941 ± 0.595^b^	1.34^a^	0.15^abc^	1.17^a^	− 0.17^bc^	0.01943
* Prevotella;s__bivia*	0.009 ± 0.013	− 1.00	− 0.72	− 0.94	0.01	0.80215
* Prevotella;s__disiens*	0.047 ± 0.080	− 4.16	− 3.05	− 3.69	− 0.08	0.83669

*HB* Hempseed bran; *HBPA* HB protein extract hydrolyzed by alcalase; *FOS* fructooligosaccharides; *BC* Blank control.

^abc^Letters indicate significant differences within a line by Tukey’s honestly significant differences (HSD) test (*p* < 0.05).

*p value indicates ANOVA test for groups comparison.

The first group of OTUs included beneficial or commensal bacteria that usually respond to prebiotics. In this group, three *Bifidobacterium* were picked showing increases on the substrates and reduction on the blank control. HB and HBPA fostered *Bif. bifidum*, but just the latter did it significantly, making this taxon grew up to the 3.30% of relative abundance (*p* < 0.05). Besides, FOS fostered even *Bif. adolescentis* (*p* < 0.05). Among *Bacteroides*, five OTUs were chosen and except *B. fragilis* were all significant (*p* < 0.05). *B. thetaiotaomicron* and *B. uniformis* were the most abundant in HBPA, HB, and FOS bioreactors at the endpoint, the first recorded top shift for HBPA reaching 7.88% of total abundance. *Parabacteroides distasonis* was found rich and significantly increased after fermentation with HBPA, HB, and FOS (*p* < 0.05), but not in the blank control (*p* > 0.05). From the class of *Lactobacillales*, significant shifts (*p* < 0.05) were seen for two *Enterococcus* and two *Lactobacillus* OTUs, that augmented with the substrates and decreased in the blank control. Interestingly, while *En. durans* was largely fostered by both HBPA, HB, and FOS, *En. faecalis* just by HBPA and HB and reduced by FOS. *Lactobacillus mucosae* and *Lb. plantarum* were represented in very low amounts at the baseline and were intensively fostered by both substrates. For example, the first reached the top quantity of 0.06% with HBPA, while the second that of 0.24% with FOS. *Faecalibacterium prausnitzii* and *Akkermansia muciniphila* were more abundant after substrates fermentation and less in the blank control, although not all significantly (*p* < 0.05).

From our results, even at the depth of the species level, it was possible to highlight the prebiotic potential of HB and on larger extend of HBPA that, similarly to FOS, fostered several taxa of beneficial bacteria. In particular, the surges in these taxa were relative to: (i) three species of health associated and SCFAs-producer *Bifidobacterium*^4^; (ii) MCFAs- and sphingolipids-producer *B. thetaiotaomicron*, succinate-producer *P. distasonis*^15,16^, and (iii) competitive excluders *Lactobacillales*, as *Lb. plantarum* and *E. durans*^17,18^. Moreover, HBPA showed to be able to foster beneficial SCFAs-producer *F. prausnitzii*^5^ and fiber-degrading *B. caccae*^19^. In comparison to HB, the better performance obtained by HBPA are due the action of alcalase that gives a product with a higher rate proteins/peptides with MW around or lower than 15 kDa, and an increased percentage of soluble proteins (10%).^2^ It is reported, for example that *Lactobacillaceae* likes peptides more than proteins, and prefers to ferment low molecular weight peptides than proteins^20^.

A second list of bacterial taxa, that changed in abundance at the endpoint, was that of opportunistic species. *Bilophila wadsworthia*, *Desulfovibrio* (pathobiontic, highly proteolytic and sulphate producers) and *Escherichia albertii,* (close relative to pathogenic species) were reduced by HBPA, HB, and FOS while increased in the blank control (*p* < 0.05). In particular HPBA performed better than HB and FOS in the containment of *Bil. wadsworthia* and *Desulfovibrio.* In details, considering these three taxa, HBPA was stronger than HB but with no significant differences in the reductions (*p* > 0.05), except for that relative to *Bil. wadsworthia* (*p* < 0.05). The ability to counteract opportunistic and enteropathogenic microbes is an essential feature of a prebiotic compounds. Particularly, these species are involved in dysbiosis of the microbiota and pathogenesis^21,22^ and were reduced in a similar study on prebiotics and vegetal fibers^5^. Thus, HBPA had superior performances than HB in limiting the development of opportunistic microbes. This evidence may contribute to explain the beneficial effects of hydrolyzed proteins. Indeed, the modulation of gut microbiota usually results from unabsorbed sugars, resistant starch, and fibers, but indigestible proteins and bioactive peptides have been proven beneficial too, such as hydrolysed proteins from soy^23^. Additionally, this feature could in part attributable to the increased release of peptides with higher antioxidant capacity when HB underwent the alcalase treatment, as well as an higher content of bioactive peptides. In fact, Setti et al.^2^ found that HBPA in comparison to HB has an in vitro antioxidant activity up to 10 times stronger. Additionally, Samaei et al.^10^ identified on HBPA 47 bioactive peptides, that for the most are short sequences of a few amino acids, potentially resistant to the gastrointestinal conditions.

The third list regards to those taxa that usually respond to vegetal fibers. The performances of HBPA that deserve merit of notion are the significant reduction of *Ruminococcus gnavus* and *R. torques,* as well as that of *Colinsella aerofaciens* and *Eggerthella lenta*, similarly to FOS (*p* < 0.05). The two *Ruminococcus* are culprits of dysbiosis associated to intestinal syndromes and are effective responders to fiber diet regime^24^. In contrast to FOS, HPBA and minorly HB were able to increase the quantity of *Oscillospira* and *Sutterella*. For HBPA the surges were significant reaching 11.90%, and 1.94% of relative abundance, respectively (*p* < 0.05). Despite the role in gut microbiota of *Oscillospira* remains enigmatic, as a member of *Ruminococcaceae* should be implicated in fiber degradation^25^ and could explain the reduced abundance observed in HPBA for same family member *F. prausnitzii*. *Sutterella*, in the past indicated as an opportunistic species has been recently reconsidered for its ability to degrade plant-based pectins and similar compounds in in vitro systems likely the MICODE^26^.

### qPCR prebiotic index

**Figure 1:**
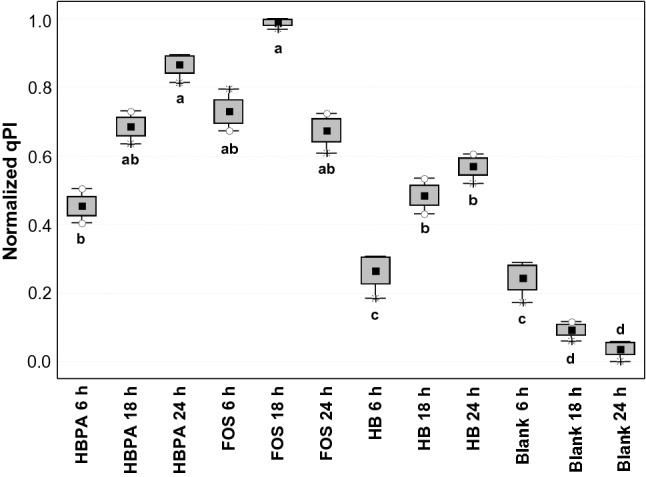
qPCR Prebiotic Index (qPI) of colonic fermentations on the substrates HBPA, HB, FOS, and on a blank control, at different time points. ^abcd^Different letters indicate statistical significance by Tukey’s honestly significant differences (HSD) test (*p* < 0.05). Marker = mean; box = mean ± S.D.; whiskers = min and max; dots = outliers; asterisks = extremes. *HB* Hempseed bran; *HBPA* HB protein extract hydrolyzed by alcalase; *FOS* fructooligosaccharides; *Blank* Blank control.

qPCR Prebiotic Index (qPI) was recently introduced^9^ as a revised method based on an updated Prebiotic Index equation, originally elaborated on 24 h controlled batch culture condition with 1% w/v addition of prebiotic by Palframan et al.^27^. Considering the results (Fig. 1), we found out that the fermented substrate with the best prebiotic activity was FOS after 18 h, and the runner-up was HBPA after 24 h. In comparison to FOS 18 h, HBPA 18 h scored 1.44-fold lower values. The blank control scored for any time points lower values than any HBPA, HB, and FOS cases (all significant, but one) and reached the lowest value of the dataset at the endpoint (26.24-fold lower than FOS 18 h).

So far, the qPI of HBPA leans to reach high level later than the FOS. Anyhow, even at the earlier time points qPI of HBPA was higher than the blank control. Thus, the comparable prebiotic index of HBPA could be mostly due to its high portion of soluble fibers. Similarly to FOS it is known that soluble fibers are excellent substrates for production of SCFAs in the large intestine^15^.

### Changes in main microbial metabolites related to prebiotic potential

**Figure 2:**
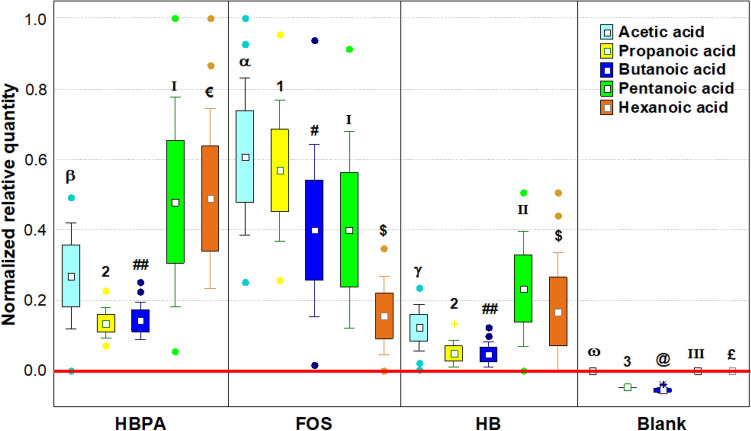
Changes in the abundance of beneficial microbial VOCs metabolites, expressed as normalized scale from relative abundances with respect to the baseline (red line). The baseline absolute quantifications in mg/kg are found in the Supplementary Material (Table S3). Changes were recorded after 6, 18, and 24 h of in vitro fecal batch fermentations with HBPA, HB, FOS, and a blank control. Each plot is made with the raw data obtained from each time point and replica. Samples were analyzed in duplicate from two independent experiments (*n* = 4). Marker = mean; box = mean ± S.D.; whiskers = non outlier range; dots = outliers; asterisks = extremes. Cases with different letters or numbers or symbols among a single independent variable are significantly different according to Tukey’s HSD test (*p* < 0.05). *HB* Hempseed bran; *HBPA* HB protein extract hydrolyzed by alcalase; *FOS* fructooligosaccharides; *Blank* Blank control.

**Figure 3:**
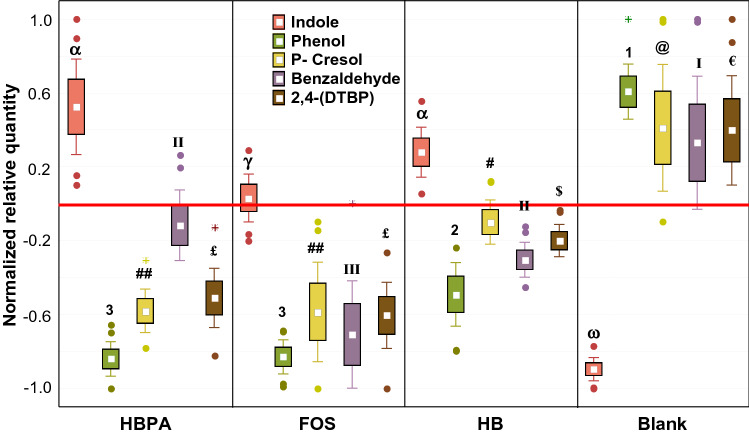
Changes in the abundance of detrimental microbial VOCs metabolites, expressed as normalized scale from relative abundances with respect to the baseline (red line). The baseline absolute quantifications in mg/kg are found in the Supplementary Material (Table S3). Changes were recorded after 6, 18, and 24 h of in vitro fecal batch fermentations with HBPA, HB, FOS, and a blank control. Each plot is made with the raw data obtained from each time point and replica. Samples were analyzed in duplicate from two independent experiments (*n* = 4). Marker = mean; box = mean ± S.D.; whiskers = non outlier range; dots = outliers; asterisks = extremes. Cases with different letters or numbers or symbols among a single independent variable are significantly different according to Tukey’s HSD test (*p* < 0.05). *HB* Hempseed bran; *HBPA* HB protein extract hydrolyzed by alcalase; *FOS* fructooligosaccharides; *Blank* Blank control.

To analyze the main changes in volatile microbial metabolites related to prebiotic potential, we have considered the shift in loads from the baseline to the endpoint (24 h) of fermentations of 10 selected VOCs (ANOVA *p* < 0.05) with renowned bioactivity in humans (short and medium chain organic acids and aromatic compounds) as follows: (a) each single compound was normalized (mean centering method) within its dataset, which included cases from HB, HBPA, FOS, and the blank control at different time points; (b) the baseline dataset (Table S4) was then subtracted to the endpoint dataset; (c) *post-hoc* analysis was done to compare the sample productions of a single molecule (Tukey’s HSD, *p* < 0.05). The first set of compounds is relative to low organic acids, such as Acetic, Propanoic, Butanoic, Pentanoic, and Hexanoic acids that are beneficial compounds essential for the host, the mucosa, and the colon microbiota itself (Fig. 2). The second set is relative to compounds related to proteolytic fermentation and/or detrimental for the host, such as Indole, Phenol, p-Cresol, Benzaldehyde, and Phenol, 2,4-bis(1,1-dimethylethyl)- (2,4-DTBP) (Fig. 3).

From the results shown in Fig. 2, organic acid concentration was increased with HB, HBPA, and FOS, while no changes nor production of any of them was recorded in the blank control. Starting from small amounts detected at the baseline (< 0.010 mg/kg for Acetic, 0.012 mg/kg for Propanoic, 0.101 mg/kg for Butanoic), the capacity to produce Acetic, Propanoic, and Butanoic acids was generally (considering the means of every time points) stronger for FOS than for HB or HBPA (*p* < 0.05). In particular, FOS fermentation accounted for 2.25-, 3.37-, and 4.87-folds more than HBPA, respectively for these three compounds. A reduction in Acetic, Propanoic, and Butanoic acids abundances is linked to dysbiosis of the colon microbiota and a reduced intestinal cell homeostasis^4^. The prebiotic activity of HBPA is linked to its capacity to foster *Lactobacillus* spp., *Bifidobacterium* ssp., and *Enterococcus* spp. that metabolize the fibers and produce low organic acids. On the opposite, starting from little amounts at the baseline (< 0.010 mg/kg either for Pentanoic and Hexanoic acids), the surge of Pentanoic and Hexanoic acids was stronger for HBPA than FOS (*p* < 0.05). In details, HBPA fermentation accounted for 1.27- and 2.08-folds more, respectively of these two compounds. Besides, HBPA fermentation released its top abundances at the endpoint, while FOS was able to release those compounds earlier, reaching the top at the intermediate time point (18 h), except for Pentanoic acid. Pentanoic and Hexanoic acids are medium chain fatty acids (MCFAs) are protective on glucose homeostasis and against insulin resistance and are important metabolic biomarkers of dysbiosis and intestinal bowel disease (IBD)^14,28,29^. The increased abundance in MCFAs observed in this study could be due to the ability of HBPA to foster *Bifidobacteriaceae* and commensals *Clostridium* group IV*,* or *Bacteroides* spp. Actually, MCFA production by these three bacterial groups happened during fiber fermentation^30^.

The ability of HBPA to liberate once fermented more SCFAs than HB could be due to the higher availability of lower MW peptides/proteins and to the higher fermentation preference of these substrates by *Lactobacillales* and *Bifidobacteriaceae*. Similarly, the more of these species were fostered and the more was the ability to elongate MCFAs from lactate production via reverse β-oxidation^31^.

The second set comprised VOCs that had a different trend for the substrates than the blank control (Fig. 3). Indole abundances increased with HB, HBPA, and FOS but decreased in BC. Oppositely, Phenol, p-Cresol, Benzaldehyde, and 2,4-(DTBP) were reduced with HBPA and partially with HB, while increased with BC. HBPA was able to produce 2.08-fold more Indole than FOS, and to reduce 1.54- and 1.48-fold more Phenol and 2,4-(DTBP) than FOS, respectively (*p* < 0.05). Indole is a tryptophan catabolite, deriving from degradation of the proteinaceous portion of the food^5^ by commensal *Escherichia coli*. Indole is also suggested to have beneficial effects, such as the attenuation of inflammation indicators on HCT-8 cells at the concentration of 1mM^32^. Otherwise, its accumulation as bacterial products (*Clostridium* spp. and *Escherichia* spp.) could result toxic for the host, because if it is not microbially degraded in beneficial derivates (*e.g.* Indole propionic acid) is metabolized into Indoxyl sulphate in the liver that, as the prototype of protein–bound uremic toxins^33^, provokes chronic kidney disease and vascular disease^5,34^. Despite, the dose of indole to generate such detrimental effect is undefined, a study finds that cattle injected with 0.2 g/kg of body weight after 72 h had diarrhoea, haemolysis, haemoglobinuria, and microscopic lesions of haemoglobinuric nephrosis^35^.

Similarly, Phenol and p-Cresol are derived from proteolytic fermentation and have been shown to damage epithelial barrier function in vitro and can be potentially carcinogenic^5^.

From the results shown in Fig. 3, FOS, HB, and HBPA fermentations indicated increases in Indole content in respect to the baseline, although significant just for HBPA (*p* < 0.05) and a reduction in metabolites (phenols) related to animal fat and protein degradation. This scenario was opposite for fermentation with the BC.

These compounds were more abundant at the baseline, as derived by fecal samples of omnivores. Their reductions are in line with the results obtained from the microbiota, indicating an increase in those taxa specialized in plant-based fibers fermentation. When comparing the reduction of these detrimental VOCs of HBPA to that of HB, the better action of HBPA could be attributed to higher bioactivity of alcalase treated HB. Indeed, a previous study demonstrated that HBPA low MW (Molecular Weight) proteins or peptides have an antioxidant activity higher than the high MW proteins or peptides of HB^2^.

### Volatilome analysis through SPME GC/MS

The SPME GC-MS analyses were conducted on 32 duplicated cases (n = 64). With NIST 11 MSMS library and the NIST MS Search program 2.0 (NIST, Gaithersburg, MD, USA) 125 molecules with more than 80% of similarity were identified, of which 77 were relatively quantified at the baseline and 113 during and after colonic fermentations. The whole volatilome was produced from a dataset of 93 significant VOCs (ANOVA at *p* < 0.05) and presented as a quantification heatmap (Figure S2). Afterwards, this dataset was separated and super-normalized by chemical classes of VOCs, i.e., organic acids, main detrimental aromatic VOCs, aldehydes, ketones, alcohols, and others (alkenes, alkanes, amines, sulphurates). Organic acids VOCs and detrimental aromatic VOCs were just previously discussed, as main microbial metabolites related to prebiotic activity, while, from each dataset of the other classes, multivariate analyses, such as untargeted Principal Component Analysis (PCA) and targeted MANOVA (*p* < 0.01) was achieved to address the specific contributes to VOCs production by the independent variables^3,8,9^. Super-normalization of the dataset was essential to unveil the effect of those compounds that are less volatile than others and could be underrepresented, as well as to avoid comparing one chemical class to another^3,8,9^.

**Figure 4:**
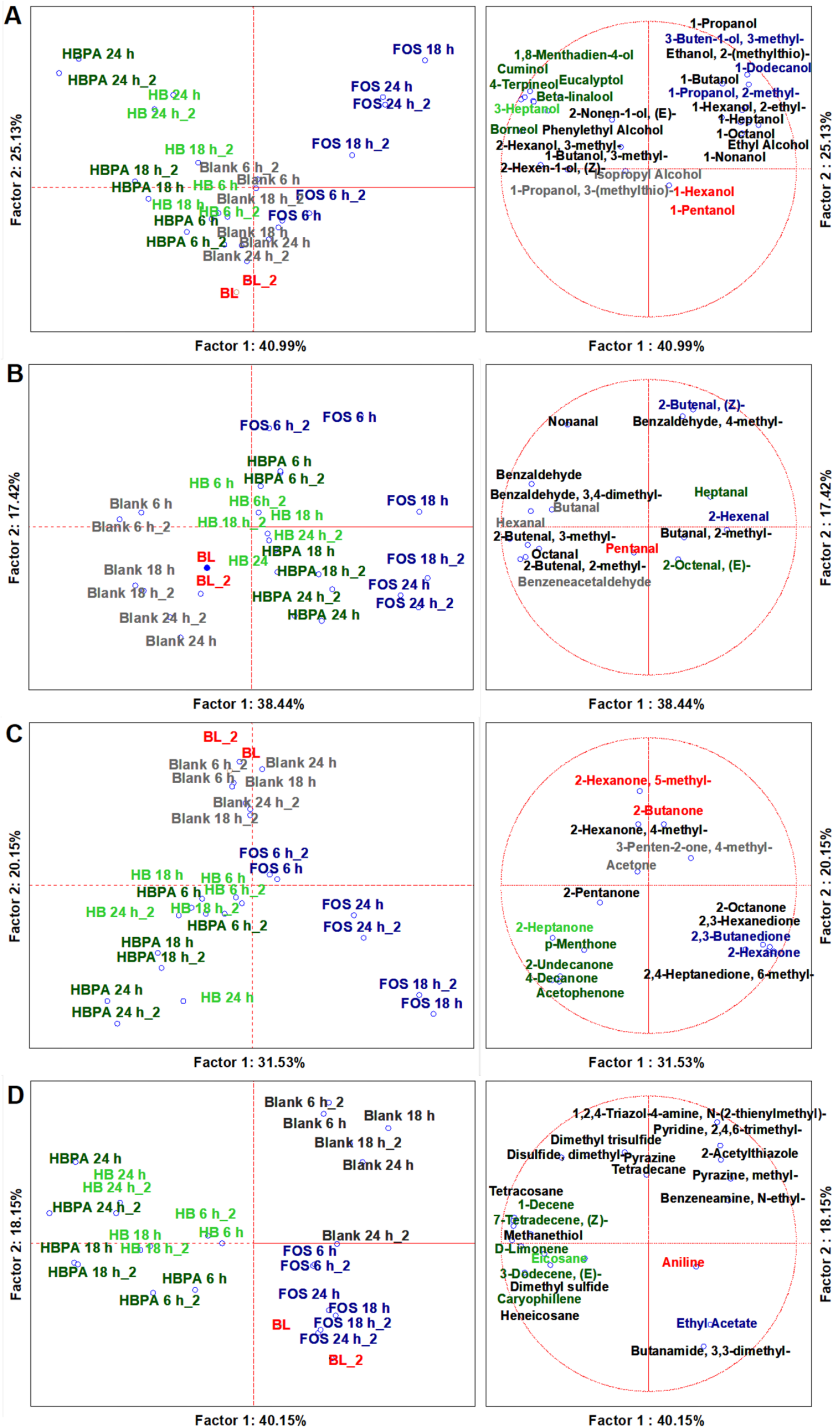
PCAs of the volatilome sorted by chemical classes of significant (ANOVA *p* < 0.05) VOCs, including the biological replicas of HBPA, HB, FOS, the blank control, and the baseline (BL) and three different timepoints (6 h, 18 h, 24 h). (**A**) Alcohols; (**B**) Aldehydes; (**C**) Ketones; (**D**) Other VOCs. Left side diagrams are for PCAs of cases; right side diagrams are for PCAs of variables. Variables with different colors are the main descriptors of the respective group of cases by MANOVA with categorical predictors as “Time Effect “ and “Matrix Effect” (Table S5, S6). *HB* Hempseed bran; *HBPA* HB protein extract hydrolyzed by alcalase; *FOS* fructooligosaccharides; *Blank* Blank control; *_2* Biological Replicates.

A PCA of 27 statistically significant alcohols distributed cases on the plot, separating fermentation with HBPA, HB, FOS, and BC from each other and from the baseline (Fig. 4A). From our results, the main descriptors of fermentation with HBPA were mainly complex terpenoid alcohols (*p* < 0.01), such as 4-Terpineol, Beta-Linalool, Cuminol, Eucalyptol, Borneol, and 1,8-Menthadien-4-ol, mainly produced at the intermediate and late time points (*p* < 0.01) while those for FOS were 1-Dodecanol, Propanol, 4-methyl, 3-Buten-1-ol, 3-methyl, and Ethyl alcohol mainly produced at the intermediate time point (*p* < 0.01) (Tables S5, S6). The main descriptor of alcohol production from BC samples remained Isopropyl alcohol (*p* > 0.01). The colon microbiota produces different alcohols during fermentation of dietary polysaccharides. Terpineol, Beta-Linalool, Cuminol, Eucalyptol, and Borneol, that are major terpenoids found in hemp seed with anti-oxidant and anti-inflammatory activity, were increased after lactobacilli fermentation of HB^3^.

Considering aldehydes, we found 16 significant VOCs that by PCA discriminated the HBPA and HB from the controls and from the baseline (Fig. 4B). The main descriptor of fermentation with FOS was 2-Hexenal (*p* < 0.01), while that for HBPA were Heptanal and 2-Octenal, (E), principally produced at the early time point and at the endpoint, respectively (*p* < 0.01) (Tables S5, S6). Lastly, the main descriptor of BC was Benzeneacetaldheyde, that was present at the baseline, but absent after fermentation with the substrates. Aldehydes are a result of microbial fermentation and lipid oxidation. Certain aldehydes are health-promoters, like 2-Octenal, (E) that was reported to limit the growth of several intestinal pathogens at a very low concentration^36^, while most are detrimental, being cytotoxic at a low threshold, such as Benzeneacetaldheyde^36^.

Considering Ketones, 16 significant VOCs were able to discriminate by PCA the substrates from each other and from the baseline (Fig. 4C). Descriptors of fermentation with HBPA were p-Menthone (77.00%) and Acetophenone (81.00%), majorly produced at the endpoint (77.04% and 51.83%, respectively) (*p* < 0.01) (Tables S5, S6). The main descriptor of fermentation with FOS was 2,3-Butadione (68.93%), that of HB and BC were 2-Heptanone and Acetone, respectively but not significantly (Table S5). During colonic fermentation, many ketones are produced; considering their bioactive attributes, some are desirable, such as Acetophenone that acts as antimicrobial to different Gram-negative bacteria, and its N-substitute derivates have been proposed as a therapeutic approach in diabetes^37^. In our experimental condition, Acetophenone is probably derived from the bacterial deconjugation of polyphenols, as *Lactobacillales*^38^, which was increased by hydrolytic process in HBPA.

A PCA of 22 statistically significant VOCs (alkenes, alkanes, amines, and sulphurates) distributed cases on the plot, separating the substrates from each other and from the baseline (Fig. 4D). The main descriptor of fermentation with FOS was Ethyl Acetate (*p* < 0.01), while those for HBPA were Caryophillene and D-Limonene, that for HB was Eicosane, while the baseline was described by Aniline. These VOCs were discriminated significantly just for the category of substrates (*p* < 0.01) (Tables S5), but no significant differences were detected for the category of time (*p* > 0.05), except for Aniline that was reduced significantly on a time basis (*p* < 0.01) (Table S6). Caryophillene and D-Limonene are potent health-related terpenes^3^ and the features observed indicate that the descriptors of HBPA were not subject to fermentation and thus their bioactivity was preserved from the food matrix. Aniline is instead a carcinogen derived from benzenoid pollutants^36^, and its reduction by fermentation with HBPA is a positive feature.

### Interomic correlations among bioactive metabolites and the microbiota

**Figure 5:**
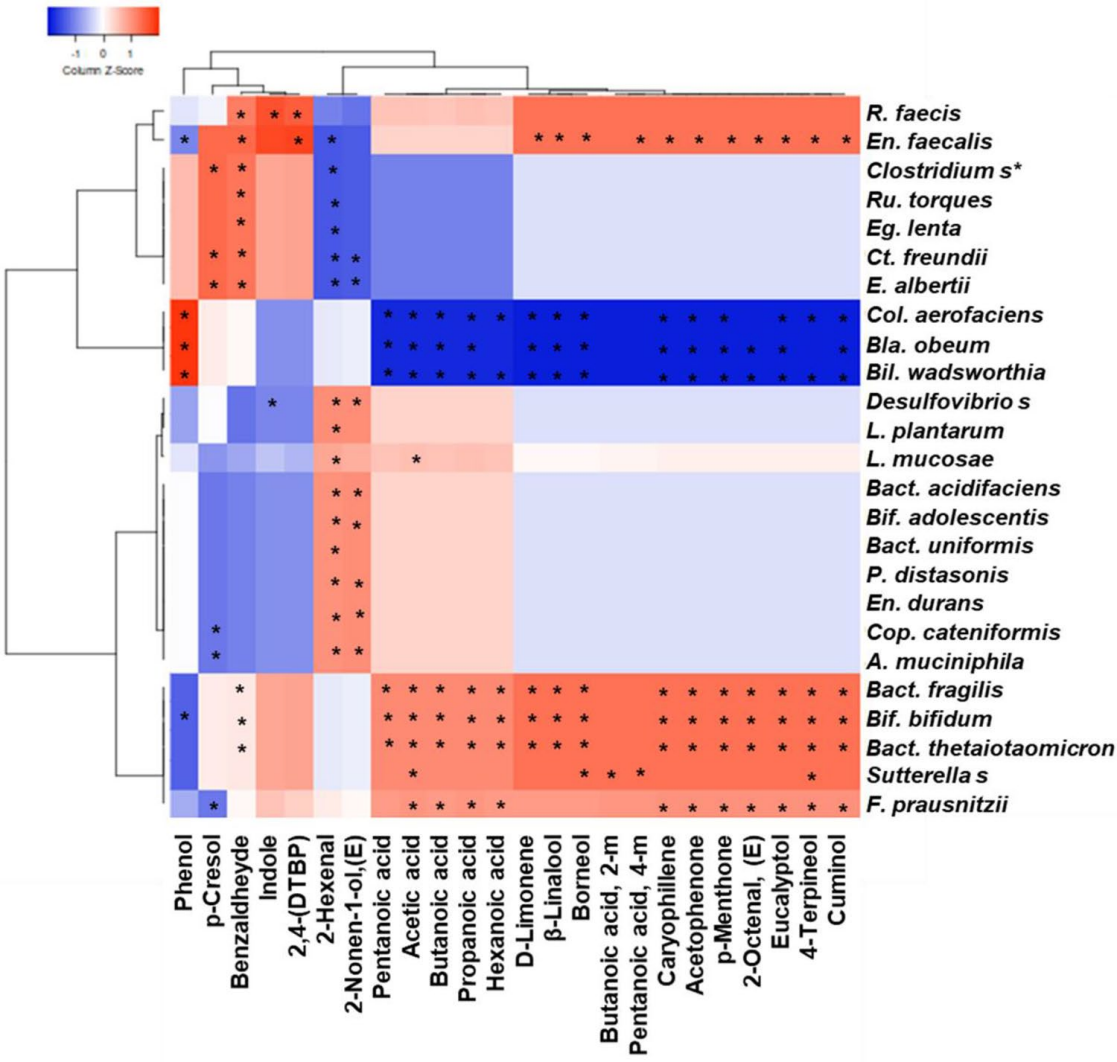
Interomics, Spearman Rank Correlations from the HBPA datasets related to microbial metabolites of the volatilome and species OTUs from the microbiota. Left side dendrogram identifies by Pearson analysis three major different clusters among bacterial species. Heat map was generated with the Expression tool on http://www.heatmapper.ca/expression/ (last accessed on 2 January 2023). Significance of correlations are provided as supplementary material (Table S7).

Spearman Rank Correlations (*p* < 0.05), two-joining-way Heatmaps, and Pearson cluster analysis were performed by the comparison of two different normalized datasets, each derived from values of relative quantification (OTUs and VOCs) of the sole HBPA dataset (Fig. 5). The significance of correlations is reported in Table S7. From the Pearson dendrograms, two main clusters and a smaller one was identified that probably may explain the cause and effect of the prebiotic potential of HBPA. The first cluster related to bacterial taxa included *Bif. bifidum, Bact. fragilis, Bact. thetaiotaomicron, Sutterella spp.,* and *F. prausnitzii,* that have positive correlations with beneficial SCFAs and MCFAs, as well as with bioactives VOCs such as 4-Terpineol, Borneol, Acetophenone and others. This cluster has also negative correlations with detrimental Phenol and p-Cresol. The second cluster included *Colinsella aerofaciens, Blautia obeum, and Bilophila wadsworthia* that have negative correlations with most of the beneficial compounds, and positive correlations with Phenol. These features have been reported by other studies, as this group of bacteria is related to dysbiosis and intestinal syndromes^39^. Lastly the third small cluster included *Roseburia faecis* and *En. faecalis*, that have positive correlations with most of the beneficial compounds and negative correlations with Phenol. Interestingly, *R. faecis* was the only one positively correlated with Indole, in line with recent findings^40^.

## Material and methods

### Fecal donors

Fecal samples were obtained and processed following previous protocols^8,9,15,41–43^. Fecal donations were obtained from three healthy subjects, two females and one male aged between 30 and 45 y. Donors did not undergo antibiotic treatment for at least 3 months prior to stool collection, did not intentionally consume pre- or probiotic supplements before the experiment, and had no history of bowel disorders. Additionally, the donors were normal weight, not smokers, not chronically consuming any drug, and not alcoholic drink consumers^8,9^. Fecal samples were donated two times (with an interval of seven days) for the two biological replicas^8,9^. To collect feces, donors were provided and instructed to use a collection kit, which includes a stool collector (Sarstedt AG & Co. KG, Nümbrecht, Germany) and an anaerobic jar with a O_2_ catalyst (Oxoid, Thermo Fisher Scientific, Waltham, MA, USA)^8,9^. Fecal samples were then maintained at 4 °C and processed within 2 h. The fecal slurry was prepared by homogenizing 6 g of feces (2 g of each donation) in 54 mL of pre-reduced phosphate-buffered saline (PBS)^8,9^. The study was conducted according to the guidelines of the ethics procedures required at the University of Bologna. Informed consent was obtained from all subjects involved in the study. The study protocol was approved by ethics committee of the University of Bologna.

### Materials

Chemicals, solvents, and enzymes for batch culture fermentation were of the highest analytical grade and were purchased from Sigma-Aldrich (St. Louis, MO, USA), Merck (Darmstadt, Germany), and Carlo Erba Reagents (CEDEX, Val de Reuil, FR), unless otherwise stated. Reagents for molecular biology and kits for DNA extraction or purifications were purchased from Thermo Fisher Scientific (USA).

### Experimental sample and controls

Experimental HB was previously prepared and characterized^2,3^. HB, the byproduct remaining after mechanical pressing of hemp seeds and subsequent grinding and sieving, was supplied by a local company (Hemp Positive World, Cesena, Italy). The original hemp variety was Futura 75. This hemp variety is registered for fiber production with a THC content < 0.2% and does not require legal permission to collect. To prepare HB samples for fermentation, 50 g of bran were resuspended in 300 mL of distilled water, sterilized (121 °C and 100 kPa for 20 min) (Vapor Matic 770, ASAL Srl, Milan, Italy) in 500 mL Corning–Pyrex bottles (Corning. NY, USA), aseptically poured on sterilized metal vessels, and stored at − 80 °C. HBPA was produced by enzymatic digestion of chemically extracted HB protein isolate, as described by Setti et al.^2^, extracting protein from HB following published procedure^44^. Briefly, the protein isolate was dissolved in deionized water (1:8, w/v) and hydrolyzed for 2 h with Alcalase (2%, v/v) at 50 °C and pH 8.0. The enzyme was then heat inactivated at 85 °C for 15 min. After cooling down to room temperature, the solution was centrifuged at 14,000 × g for 10 min, and the supernatant was collected and stored at − 80 °C.

Both HB and HPBA solutions were freeze dried with a Savant freeze-dryer Lyolab 3000 apparatus (Thermo Fisher Scientific, USA), and the powder was used to test the prebiotic potential^2,3^.

### Fecal batch-culture fermentation and samples collection

Colonic fermentations were conducted for 24 h in independent vessels on 1% (w/v) of HB, on 1% (w/v) of HBPA, on 1% (w/v) of fructo-oligosaccharides (FOS) from chicory (positive control), and on a blank control (BC) (negative control), using an in vitro gut model, MICODE (Multi-Unit in vitro Colon Model), obtained by the assembly of Minibio Reactors (Applikon Biotechnology BV, Delft, NL) and controlled by Lucullus PIMS software (Applikon Biotechnology BV, NL)^8,9^. The preparation of the experiments were made according to published procedures^5,8,9,41,42^. In details, bioreactors were autoclaved at 121 °C and 100 kPa for 15 min and once cooled aseptically filled with 90 mL of anaerobic pre-sterilized basal nutrient medium. Basal medium (BM) contained (per liter): 2 g peptone, 2 g yeast extract, 0.1 g NaCl, 0.04 g K_2_HPO_4_, 0.04 g KH_2_PO_4_, 0.01 g MgSO_4_·7H_2_O, 0.01 g CaCl_2_·6H_2_O, 2 g NaHCO_3_, 2 mL Tween 80, 0.05 g Hemin dissolved in 1 mL of 4 M-NaOH, 10 mL vitamin K, 0.5 g L-cysteine HCl, and 0.5 g bile salts (sodium glycocholate and sodium taurocholate)^41,42^. The medium was adjusted to pH 7.0 before autoclaving and 2 mL of 0.025% (w/v) resazurin solution were added afterwards once the media was cooled^41,42^. Fermentation vessels were filled aseptically with 90 mL of BM and the bioreactor headplates were mounted, including previously sterilized and calibrated sensors, i.e. pH and DO_2_ (Dissolved Oxygen) sensors. Anaerobic condition (0.0–0.1% w/v of DO_2_) in each bioreactor was obtained in about 30 min flushing with filtered O_2_-free N_2_ through the mounted-in sparger of Minibio reactors (Applikon Biotechnology BV, NL), and was constantly kept over the experiment. Temperature was set at 37 °C and stirring at 300 rpm, while pH was adjusted to 6.75 and kept throughout the experiment with the automatic addition of filtered NaOH or HCI (0.5 M) to mimic the conditions located in the distal region of the human large intestine^8,9^. Once the exact environmental settings were reached, the four vessels were aseptically injected with 10 mL of fecal slurry (10% w/v of human feces to a final concentration of 1%, w/v) and then three of them independently with 1 g of HB, HBPA or FOS (to a final concentration of 1%, w/v), while the fourth vessel was set as blank control (BC, basal medium and 1% fecal slurry only). Batch cultures were run under these controlled conditions for a period of 26.55 h during which samples were collected at 4 time points (Baseline, 6, 18, and 24 h). The baseline (BL) was defined on the first pH changes detected by Lucullus (1 read/10 s) via the pH Sensors of MICODE^8,9^. For this work, the BL was set after 2.55 ± 0.11 h. Sampling was performed with a dedicated double syringe filtered system (Applikon Biotechnology BV, NL) connected to a float drawing from the bottom of the vessels without perturbing or interacting with the bioreactor’s ecosystem. To guarantee a close control, monitoring and recording of fermentation parameters the software Lucullus 3.1 (PIMS, Applikon Biotechnology BV, NL) was used. This also allowed to keep the stability of all settings during the experiment^8,9^. Fermentations were conducted in duplicate independent experiments, using for each a new pool of feces from the same three healthy donors^8,9^.

The freeze-dried samples were directly fermented in the colon with no gastric phase digestion, as the nature of prebiotic is to reach the colon to feed the microbiota without being affected by host’s enzymes^45^.

### Pipeline of experimental activities

Parallel and independent vessels for FOS, HB, HBPA, and BC were run for 24 h after the adaptation of the fecal inoculum, defined as the baseline (BL). The entire experiment consisted of 32 cases (n = 32), including 4 theses (FOS, HB, HBPA, and BC) and 4 time points (BL, 6 h, 18 h, and 24 h) in duplicate. Samples of the different time points were used for qPCR and SPME GC-MS analyses. After sterile sampling of 5 mL of bioreactor contents, samples were centrifuged at 16,000 × g for 7 min to separate the pellets and the supernatants, which were used for bacterial DNA extraction and SPME-GC-MS analysis, respectively^8,9^. Specifically, microbial DNA extraction was conducted just after sampling so as not to reduce *Firmicutes* content^8,9^. Sampling from DNA samples and SPME-GC-MS samples were then stored at − 80 °C. Technical replicas of analyses were conducted in duplicate for SPME GC-MS (n = 64) and in triplicate for qPCR (n = 96), both from two independent experiments^8,9^.

## Microbiota related analyses

### DNA extraction, amplification and sequencing

DNA was extracted from the fecal samples (from donors and the pool) and from the MICODE effluates at each time points (BL, 6 h, 18 h, and 24 h) using the Purelink Microbiome DNA Purification Kit (Invitrogen, Thermo Fisher Scientific, Carlsbad, CA, USA)^8,9^. Nucleic acid purity was tested on BioDrop Spectrophotometer (Biochrom Ltd., Cambridge, UK). Samples from the feces, the BL, and the end point were used for MiSeq sequencing (Illumina Inc, San Diego, CA, USA), while samples from the BL and other time points were used for quantitative PCR (qPCR) analyses^8,9^. Considering the MiSeq approach, bacterial diversity was obtained by the library preparation and sequencing of the 16S r-DNA gene^8,41,42^. The following two amplification steps were performed: an initial PCR amplification using 16S locus-specific PCR primers (16S-341F 5′-CCTACGGGNGGCWGCAG-3′ and 16S-805R 5′-GACTACHVGGGTATCTAATCC-3′) and a subsequent amplification integrating relevant flow-cell-binding domains (5′-TCGTCG GCAGCGTCAGATGTGTATAAGAGACAG-3′ for the forward primer and 5′-GTCTCGTGGGCTCGGAGATGTGTATAAGAGACAG-3′ for the reverse overhang), and lastly unique indices selected among those available Nextera XT Index Kits were combined according to manufacturer’s instructions (Illumina Inc, USA)^8,46^. Both input and final libraries were quantified by Qubit 2.0 Fluorometer (Invitrogen, USA). In addition, libraries were quality-tested by Agilent 2100 Bioanalyzer High Sensitivity DNA assay (Agilent technologies, Santa Clara, CA, USA). Libraries were sequenced in a MiSeq (Illumina Inc, USA) in the paired end with 300-bp read length^46^. Sequencing was conducted by IGA Technology Service Srl (Udine, Italy).

### Sequence data analysis

Reads were de-multiplexed based on Illumina indexing system. Sequences were analyzed using QIIME 1.5.0^47^. After filtering based on read quality and length (minimum quality = 25 and minimum length = 200), Operational Taxonomic Units (OTUs) defined by a 97% of similarity were picked using the Uclust v1.2.22 q method^48^ and the representative sequences were submitted to the RDP classifier^21^ to obtain the taxonomy assignment and the relative abundance of each OTU using the Greengenes 16S rRNA gene database^49^. Alpha- and beta-diversity analyses were performed using QIIME 1.5.0^8,46^.

### Enumeration of bacterial groups for the qPI (Quantitative Prebiotic Index)

Changes in Eubacteria kingdom, *Lactobacillales* order*, Bifidobacteriaceae*, *Enterobacteriaceae* families, and *Clostridium* group I were also assessed by qPCR and SYBR Green I chemistry^9,13,50^, targeting small fragments of monocopies, or multicopy genes by degenerated or specific primer pairs, previously amplified by high-fidelity DNA polymerase (Invitrogen Platinum SuperFi II DNA Polymerase, Thermo Fischer Scientific, USA). Extraction of bacterial DNA was obtained with Pure Link Microbiome DNA Purification Kit (Invitrogen, USA). Genetic standards were prepared from relative PCR amplicons of the target bacterial species, using GeneJet Genomic DNA purification kit (Thermo Fisher Scientific, USA), as described previously^8,51^. For each of the targets, general qPCR reactions were set as follows: a holding stage at 98 °C for 6 min, and a cycling stage made of 95 °C for 20 s and 60 °C for 60 s, repeated for 45 times, followed by melting curve analysis^3,9^. Quantifications were made by a RotorGene 6000 (Qiagen, Hilden, Germany) with a five-point standard of the given amplicon, separately. Reactions were prepared with 1 ng of DNA, 2 × Power-Up SYBR Green (Thermo Fisher Scientific, USA) and 250 nM of each primer (Eurofins Genomics, Ebersberg, Germany)^3,9^. Details of primer pairs for PCR and qPCR are supplied as Table S1. All results were expressed as mean values obtained from triplicates from two independent experiments.

The Prebiotic Index was revised from the original equation elaborated by Palframan et al.^27^ introducing substitution on bacterial taxa, the molecular approach based on quicker qPCR, data normalization, sextuplicate values, and significant differences^9^. Analogously to the original method, we employed an equation based on quantification values expressed as Log_10_ cell/mL, and similar conditions applied in fermentation (24 h controlled batch with 1% w/v of prebiotic fiber). So far, the new equation for the Prebiotic Index is based on qPCR data (qPCR Prebiotic Index—qPI) as follows:

$$ \begin{aligned}   {\text{qPI}} &  = \left( {Bifidobacteriaceae/{\text{Eubacteria}}} \right) - \left( {Enterobacteriaceae/{\text{Eubacteria}}} \right) \\     & \quad  + \left( {Lactobacillales/{\text{Eubacteria}}} \right) - \left( {ClostridiumgroupI/{\text{Eubacteria}}} \right). \\  \end{aligned}  $$.

### Volatilome analysis

Volatile organic compound (VOCs) evaluation was carried out on an Agilent 7890A Gas Chromatograph (Agilent Technologies, Santa Clara, CA, USA) coupled to an Agilent Technologies 5975 mass spectrometer operating in the electron impact mode (ionization voltage of 70 eV) equipped with a Chrompack CP-Wax 52 CB capillary column (50 m length, 0.32 mm ID) (Chrompack, Middelburg, The Netherlands)^3,8,9^. The solid phase micro-extraction (SPME) GC-MS protocol and the identification of volatile compounds were done according to previous reports, with minor modifications^8,52–54^. Briefly, 3 mL of vessel content or fecal slurry were placed into 10 mL glass vials and added to 10 μL of the internal standard (4-methyl-2-pentanol) to a final concentration of 4 mg/L. Samples were then equilibrated for 10 min at 45 °C. SPME fiber, coated with carboxen-polydimethylsiloxane (85 μm), was exposed to each sample for 40 min. Preconditioning, absorption, and desorption phases of SPME–GC analysis, and all data-processing procedures were carried out according to previous publications^8,53,54^. Briefly, before each head space sampling, the fiber was exposed to the GC inlet for 10 min for thermal desorption at 250 °C in a blank sample. The samples were then equilibrated for 10 min at 40 °C. The SPME fiber was exposed to each sample for 40 min, and finally the fiber was inserted into the injection port of the GC for a 10 min sample desorption. The temperature program was: 50 °C for 1 min, then programmed at 1.5 °C/min to 65 °C, and finally at 3.5 °C/min to 220 °C, which was maintained for 25 min. Injector, interface, and ion source temperatures were 250, 250, and 230 °C, respectively. Injections were carried out in split-less mode and helium (3 mL/min) was used as a carrier gas. Identification of molecules was carried out by searching mass spectra in the available databases (NIST 11 MSMS library and the NIST MS Search program 2.0 (NIST, Gaithersburg, MD, USA). Each VOC was relatively quantified in percentage (LOD = 0.001 mg/kg)^3,8,9,55^. Besides, in samples prior to in vitro colonic fermentation (baseline) (Table S3) the main microbial metabolites related to prebiotic activity were also absolutely quantified in mg/Kg (LOQ = 0.03 mg/kg and LOD = 0.01 mg/kg)^3,8,9,53,54^. For these latter compounds, samples at the endpoint (24 h) were compared to the baseline and values were expressed as shifts. All results were expressed as normalized mean values obtained from duplicates in two independent experiments^8,9^.

### Data processing and statistical analysis

For the volatilome, one-way ANOVA (*p* < 0.05) was used to determine significant VOCs among the dataset, which included 8000 interactions generated between 125 dependent variables (VOCs) and 64 independent variables (2 technical and 2 experimental replicas of 4 different fermentation treatments; HBPA, HB, FOS, and blank control, and 4 different time points; Baseline, 6, 18, and 24 h). Also, prior ANOVA, Normality and Homoscedasticity were tested by Shapiro–Wilk’s W Test and Levene’s Test, respectively (Tables S8, S9). The significant VOCs (n = 93) represented the total volatilome of the experiments and was reported as a quantification heatmap (Figure S2). Then, from this dataset the VOCs were divided in three groups, and analyzed differently: (i) the prebiotic related VOCs (preVOCs); (ii) the detrimental VOCs and (iii) the remaining volatilome. The analyses conducted were Principal Component Analysis (PCA) to distribute the results on a plane; Multivariate ANOVA (MANOVA) to address specific contributes by categorical predictors; Student’s t-test to compare a sample to another within the same variable.

For the microbiota, after ANOVA for group comparison (the baseline versus the end point), the significant variables (*p* < 0.05) were selected and the shifts in abundance were calculated as Log_2_(F/C). Then, post hoc Tukey HSD test (*p* < 0.05) was performed on the raw data to define differences among treatments. The microbiota at the endpoint was analyzed as a pool of DNA of the biological replicas for each case, while at the baseline as a pool of the four cases. For the qPI values a dataset of 5 dependent variables (bacterial taxa) and 96 independent variables (3 technical and 2 experimental replicas of 4 different fermentation treatments, and 4 different time points, Baseline, 6, 18, and 24 h) was studied for statistical differences among time points and treatments by ANOVA (*p* > 0.05) and post hoc Tukey HSD test (*p* < 0.05). Also, prior ANOVA, Normality and Homoscedasticity were tested by Shapiro–Wilk’s W Test and Levene’s Test, respectively (Tables S10, S11). To address specific correlations among bacteria and molecules (preVOCs) and explain the prebiotic potential of HBPA, two independent HBPA datasets were merged and computed by Spearman Rank analysis and visualized with a two-way joining heatmap including Pearson dendrograms with complete linkage^8,9^. The baselines of values for the volatilome and for the microbiota were that obtained sampling just after adaptation of the microbiota to the bioreactor condition^9^. Normalization of datasets was performed with the mean centering method. Statistics and graphics were made with Statistica v.8.0 (Tibco, Palo Alto, CA, USA), but two ways joining heatmaps were performed with Expression tool on http://www.heatmapper.ca/expression/ (last accessed on 2 January 2023).

## Conclusion

Based on the positive results obtained by different beneficial (F/B ratio, microbial diversity, organic acids) or harmful (Phenol, p-Cresol, etc.) indicators, our study evidenced that HB and in particular HBPA had a prebiotic potential comparable to that of FOS. Furthermore, HBPA and minorly by HB during colonic fermentations were able to foster the populations of beneficial and fiber degrading bacteria and to contain the populations of opportunistic and proteolytic bacteria. Additionally, alcalase treatment of HB makes a product more potent, in terms of prebiotic activity probably due to a higher release of small peptides that along with being more bioactive directly on the host (i.e. antioxidant and antihypertensive) are also more accessible and specific as substrates for the fermentation by beneficial microbes, and nasty or even toxic for the fermentation by opportunistic microbes.

The use of MICODE, a robust and versatile in vitro model, together with multivariate statistics visibly demonstrated a suitable approach to describe the effects generated by the alcalase hydrolysis and to explain the prebiotic potential of hydrolysates.

Such in vitro approach could be included in a pipeline of experiments where a reduced number of animals for testing is employed, according to the Directive 2010/63/EU and the Regulation (EU) 2019/1010. To fully understand the efficacy of HBPA on human health a diet intervention study is imperative, and the results presented are target-effective and should have robustness for pre-clinical applications.

## Supplementary Information

Supplementary Information.

## Data Availability

The MiSeq 16S microbiota datasets generated and/or analyzed during the current study are available in the NCBI repository, https://www.ncbi.nlm.nih.gov/bioproject/811443. BL = SAMN26398950 on SUB11151745 with SRR18212552; BL2 = SAMN26401655 on SUB11151742 with SRR18212507; Pool = SAMN26394592 on SUB11151738 with SRR18212505; BC = SAMN26389858 on SUB11151732 with SRR18212504; FOS = SAMN26382733 on SUB11151715 with SRR18212497; HBPA = SAMN26379205 on SUB11151675 with SRR18212398; HB = SAMN26343431 on SUB11151777 with SRR18183901. Other data should be requested to lorenzo.nissen@unibo.it.
