# Supplementary material for: Beneficial metabolic transformations and prebiotic potential of hemp bran and its alcalase hydrolysate, after colonic fermentation in a gut model
Source: Sci Rep. 2023 Jan 27;13:1552. doi: 10.1038/s41598-023-27726-w (PMC9883387; doi:10.1038/s41598-023-27726-w)
Supplement: Supplementary file 1 — Supplementary Information. [file 41598_2023_27726_MOESM1_ESM.docx]

**Beneficial metabolic transformations and prebiotic potential of hemp bran and its alcalase hydrolysate. after colonic fermentation in a gut model**

**Lorenzo Nissen^1.2^, Flavia Casciano^1^, Elena Babini^1.2^, Andrea Gianotti*^1.2^**

^1^ Department of Agricultural and Food Sciences (DISTAL). *Alma Mater Studiorum* - University of Bologna. Piazza Goidanich 60, 47521 Cesena, Italy.

^2^ Interdepartmental Centre of Agri-Food Industrial Research (CIRI). *Alma Mater Studiorum* - University of Bologna. Piazza G. Goidanich 60, 47521 Cesena, Italy.

*Corresponding author: [andrea.gianotti@unibo.it](mailto:andrea.gianotti@unibo.it)

**Supplementary Materials**

**Table S1.** Primers pairs employed for PCR and qPCR reactions and quantifications.

**Table S2.** Abundances (% ± S.D.) and changes in phylum taxa

**Table S3.** Abundances (% ± S.D.) and changes in family taxa (Log_2_ F/C) after 24 h in vitro colonic fermentations from healthy donors and administrated with HBPA, HB, and FOS as the substrates.

**Table S4.** Quantification of VOCs by SPME GC/MS related to prebiotic potential. employing 10000 mg/kg of 2-Pentanol, 4-methyl.

**Table S5.** MANOVA categorical descriptors for the volatilome. categorized for the type of matrix. % of contribution of VOCs descriptors significant among the food matrices.

**Table S6.** MANOVA categorical descriptors for the volatilome. categorized for the time of fermentation. % of contribution of VOCs descriptors significant among the time points.

**Table S7.** Significance of Spearman Rank correlations

**Table S8.** Wilk Shapiro W test for Normality of the volatilome

**Table S9.** Levene’s Test for Homoscedasticity of the volatilome

**Table S10**. Wilk Shapiro W test for Normality of the Microbiota at the species level

**Table S11**. Levene’s Test for Homoscedasticity of the Microbiota at the species level

**Figure S1. A – F**. Plots of Alpha Diversity indices. A = Observed OTU index for microbiota richness; B = Shannon index for microbiota evenness; C = Simpson index for microbiota dominance; D = Chao 1 index for microbiota abundance; E = Good’s index for microbiota rarity; F = Bray Curtis PCoA of Beta Diversity. Red dots = duplicates of stool microbiota from donors (D1 – D3); orange dot = duplicate of pooled stool microbiota from donors; pale blue dot = duplicates of baseline microbiota from bioreactors; blue dot = duplicates of endpoint microbiota of FOS fermentations; pale green dot = duplicates of endpoint microbiota of HB fermentations; green dot = duplicates of endpoint microbiota of HBPA fermentations; black dot = duplicates of endpoint microbiota of HBPA fermentations; ^abc^Different letters indicate significance (*t*-student *p* < 0.05) within a plot.

**Figure S2.** Quantification heatmap of total VOCs.

**Table S1.** Primers pairs employed for PCR and qPCR reactions and quantifications.

| **Group** | **Target** | **Sequence 3’-5’** | **Bp*** | **Reference** |
| --- | --- | --- | --- | --- |
| *Eubacteria* | V3-V4  16 S | Eub518-R: ATTACCGCGGCTGCTGG | 147 | 1 |
|  |  | Eub338-R: ACTCCTACGGGAGGCAG |  |  |
| *Enterobacteriaceae* | V3-V4  16 S | Enterobac-F: TGCCGTAACTTCGGGAG | 450 | 2 |
|  |  | Enterobac-R: TCAAGGACCAGTGTTCAG |  |  |
| *Lactobacillales* | V3-V4  16 S | Lac-F: GCAGCAGTAGGGAATCT | 340 | 3 |
|  |  | Lac-R: GCATTYCACCGCTACACA |  |  |
| *Bifidobacteriaceae* | RecA | RecA-F: CGTYTCBCAGCCGGAYA | 220 | 4 |
|  |  | RecA-R: CCARVGCRCCGGTCATC |  |  |
| *Clostridium* group I | V3-V4  16 S | ClosI-F: TACCHRAGGAGGAAGCCAC | 148 | 2 |
|  |  | ClosI-R: GTTCTTCCTAATCTCTACGCAT |  |  |

*Base pairs.

**Table S2.** Abundances (% ± S.D.) and changes in phylum taxa (Log_2_ F/C) after 24 h *in vitro* fecal batch culture fermentations from healthy human donors with HBPA, HB, and FOS as the substrates.

|  | **% Relative abundance** | **Log_2_(F/C) changes at the end points (24 h)** | | | |  |
| --- | --- | --- | --- | --- | --- | --- |
| **Taxon** | **Baseline Mean** | **HBPA** | **FOS** | **HB** | **BC** | ***p* value*** |
| Unclassified;Other | 0.036 ± 0.013^b^ | 0.98^b^ | 2.27^a^ | 0.48^b^ | -1.40^c^ | 0.00446 |
| *Archaea*;Other | 0.002 ± 0.002 | 1.29 | 0.09 | 0.29 | n.d. | 0.17606 |
| *Euryarchaeota* | 0.485 ± 0.420 | -7.33 | -8.52 | -5.38 | -8.46 | 0.46025 |
| *Bacteria;Other* | 0.107 ± 0.035 | 0.66 | 0.02 | 0.26 | -1.38 | 0.16544 |
| *Actinobacteria* | 8.392 ± 2.848 | 0.60 | 0.98 | 0.45 | -0.07 | 0.25278 |
| *Bacteroidetes* | 32.634 ± 8.671^a^ | -0.19^a^ | 0.33^a^ | -0.27^a^ | -2.14^b^ | 0.00476 |
| *Firmicutes* | 54.206 ± 10.431^a^ | -0.30^a^ | -0.84^b^ | -0.32^a^ | -0.48^a^ | 0.03891 |
| *Proteobacteria* | 3.166 ± 0.423^d^ | 2.07^b^ | 1.68^c^ | 1.07^b^ | 3.82^a^ | 0.00006 |
| *Verrucomicrobia* | 0.903 ± 0.987 | 0.29 | 0.55 | 0.21 | -4.78 | 0.32048 |
| F/B** | 1.662 ± 0.071^b^ | -0.10^b^ | -1.08^c^ | -0.04^b^ | 2.84^a^ | 0.00615 |

* *p* value indicates ANOVA test for groups comparison; ^abc^ Letters indicate significant differences within a taxon by Tukey’s honestly significant differences (HSD) test (*p* < 0.05); **F/B *= Firmicutes/Bacteroidetes*; HB = Hempseed bran; HBPA = HB protein extract hydrolyzed by alcalase; FOS = fructooligosaccharides; BC = Blank control.

**Table S3**. Abundances (% ± SD) and changes in family taxa (Log_2_ F/C) after 24 h *in vitro* colonic fermentations from healthy donors and administrated with HBPA, HB, and FOS as the substrates.

| **Taxon** | **% Relative abundance** | **Log_2_(F/C) changes** | | | | ***p* value*** |
| --- | --- | --- | --- | --- | --- | --- |
|  | **Baseline Mean** | **HBPA 24 h** | **FOS 24 h** | **HB 24 h** | **BC 24 h** |  |
| Unclassified | 0.052 ± 0.013^b^ | 0.49^b^ | 1.77^a^ | 0.40^b^ | -1.91^c^ | 0.02944 |
| *Archaea*;Other | 0.002 ± 0.002 | 1.68 | 0.48 | 1.05 | n.d. | 0.32766 |
| *Methanobacteriaceae* | 0.002 ± 0.420 | 0.68 | -0.52 | 0.55 | -0.45 | 0.64868 |
| *Bacteria*;Other | 0.078 ± 0.035 | 1.13 | 0.48 | 0.98 | -0.92 | 0.31403 |
| *Actinomycetaceae* | 0.014 ± 0.002^a^ | -0.90^b^ | n.d. | -0.80^b^ | -0.77^b^ | 0.04404 |
| *Corynebacteriaceae* | 0.006 ± 0.006 | -1.90 | -2.10 | -1.77 | -2.04 | 0.83981 |
| *Bifidobacteriaceae* | 8.143 ± 2.844 | 0.65 | 1.03 | 0.55 | -0.11 | 0.05389 |
| *Bacteroidetes*;Other | 0.002 ± 0.017 | 3.27 | 1.81 | 3.05 | n.d. | 0.74702 |
| *Bacteroidales*;Other | 0.020 ± 0.007 | n.d. | -3.91 | n.d. | n.d. | 0.47719 |
| *Bacteroidaceae* | 29.267 ± 6.622^a^ | -0.37^a^ | 0.23^a^ | -0.30^a^ | -3.15^b^ | 0.04929 |
| *Odoribacteraceae* | 0.137 ± 0.081 | -5.51 | -4.70 | -5.13 | -5.64 | 0.22275 |
| *Porphyromonadaceae* | 2.315 ± 0.720 | 0.53 | 1.45 | 0.44 | 0.78 | 0.05591 |
| *Prevotellaceae* | 0.063 ± 0.032 | -1.59 | n.d. | -1.45 | n.d. | 0.84834 |
| *Rikenellaceae* | 2.765 ± 0.820 | -1.03 | -2.03 | -0.97 | -5.00 | 0.31813 |
| *Sphingobacteriaceae* | 0.008 ± 0.043 | -2.49 | n.d. | -2.34 | -1.04 | 0.69161 |
| *Firmicutes*;Other | 0.011 ± 0.102 | n.d. | n.d. | n.d. | 0.42 | 0.66869 |
| *Gemellaceae* | 0.003 ± 0.001 | n.d. | n.d. | n.d. | n.d. | 0.43166 |
| *Enterococcaceae* | 0.011 ± 0.668^b^ | 8.76^a^ | 9.60^a^ | 8.50^a^ | 5.88^a^ | 0.02150 |
| *Lactobacillaceae* | 0.161 ± 0.081 | 0.50 | 0.94 | 0.23 | -1.35 | 0.07355 |
| *Leuconostocaceae* | 0.004 ± 0.002 | n.d. | n.d. | n.d. | -1.45 | 0.30679 |
| *Streptococcaceae* | 0.729 ± 0.148 | -2.19 | -4.87 | -1.56 | -1.51 | 0.58467 |
| *Clostridia*;Other | 0.004 ± 0.015 | 0.27 | nd | 0.12 | -1.45 | 0.59937 |
| *Clostridiales*;Other | 0.208 ± 1.208 | -3.11 | -1.88 | -2.65 | -1.22 | 0.59476 |
| *Clostridiaceae* | 0.363 ± 0.125^c^ | 5.32^a^ | 2.09^b^ | 5.05^a^ | 5.73^a^ | 0.00339 |
| *Eubacteriaceae* | 0.012 ± 0.003 | -1.43 | -1.22 | -0.98 | -0.83 | 0.52272 |
| *Lachnospiraceae* | 23.207 ± 4.802^a^ | -2.55^b^ | -1.36^b^ | -2.01^b^ | -1.72^b^ | 0.04585 |
| *Peptococcaceae* | 0.027 ± 0.015 | n.d. | n.d. | n.d. | -4.31 | 0.66121 |
| *Peptostreptococcaceae* | 2.497 ± 1.090 | -0.09 | -2.55 | -0.03 | 0.12 | 0.56452 |
| *Ruminococcaceae* | 10.021 ± 5.956 | 0.64 | -1.69 | 0.32 | -2.63 | 0.22851 |
| *Veillonellaceae* | 8.867 ± 2.141 | -5.30 | -0.95 | -5.01 | -2.21 | 0.23552 |
| *Coriobacteriaceae* | 4.771 ± 0.971 | -2.07 | -2.53 | -1.71 | 0.12 | 0.05386 |
| *Erysipelotrichales*;Other | 0.019 ± 0.017 | n.d. | n.d. | n.d. | -0.77 | 0.65062 |
| *Coprobacillaceae* | 0.380 ± 0.208 | 1.24 | 1.67 | 0.87 | -2.44 | 0.05659 |
| *Erysipelotrichaceae* | 0.191 ± 0.143 | -0.61 | 0.99 | -0.24 | -3.53 | 0.52436 |
| *Alphaproteobacteria*;Other | 0.073 ± 0.035 | -3.60 | -5.80 | -3.02 | -4.15 | 0.78999 |
| *Rhodospirillales*;Other | 0.173 ± 0.090 | n.d. | -4.23 | n.d. | -2.80 | 0.87420 |
| *Alcaligenaceae* | 1.661 ± 0.596 | 1.57 | 0.92 | 0.91 | -0.94 | 0.07159 |
| *Desulfovibrionaceae* | 0.165 ± 0.054 | -3.19 | 1.06 | -3.00 | -0.80 | 0.04846 |
| *Enterobacteriaceae* | 0.529 ± 0.103^b^ | 4.15^a^ | 3.66^a^ | 4.03^a^ | 6.37^a^ | 0.00002 |
| *Pseudomonadaceae* | 0.004 ± 0.001 | -0.32 | nd | -0.22 | nd | 0.12048 |
| *Verrucomicrobiaceae* | 0.904 ± 0.330^a^ | 0.30^a^ | 0.56^a^ | 0.05^a^ | -4.82^b^ | 0.04306 |

**p* value indicates ANOVA test for groups comparison; ^abcd^ Different letters indicate statistical significance by Tukey’s HSD test (*p* < 0.05); n.d. = not detected; HB = Hempseed bran; HBPA = HB protein extract hydrolyzed by alcalase; FOS = fructooligosaccharides; BC = Blank control.

**Table S4.** Quantification of VOCs by SPME GC/MS related to prebiotic potential, employing 10000 mg/kg of 2-Pentanol, 4-methyl.

| **VOCs** | **mg/kg ± S.D.** | ***p* value^†^** |
| --- | --- | --- |
| Acetic acid | traces* | 0.0010 |
| Propanoic acid | 0.012 ± 0.015 | 0.0467 |
| Butanoic acid | 0.101 ± 0.086 | 0.0424 |
| Pentanoic acid | traces | 0.0153 |
| Hexanoic acid | traces | 0.0179 |
| Indole | 7.955 ± 1.388 | <0.0001 |
| Phenol | 0.177 ± 0.051 | <0.0001 |
| Phenol, 4-methyl- | 15.022 ± 9.808 | 0.0141 |
| Benzaldehyde | 0.717 ± 0.415 | 0.0013 |
| 2.4-(DTBP)** | 1.826 ± 0.624 | 0.0074 |

*traces < 0.01 mg/kg; **Phenol, 2,4-bis(1,1-dimethylethyl)-; (LOQ = 0.03 mg/kg and LOD = 0.01 mg/kg); **^†^** *p* value of ANOVA from a dataset including all cases and time points.

**Table S5**. MANOVA categorical descriptors for the volatilome categorized for the type of matrix. % of contribution of VOCs descriptors significant among the food matrices.

| **VOC** | | **Baseline** | **HBPA** | **FOS** | **HB** | **BC** | ***p* value*** |
| --- | --- | --- | --- | --- | --- | --- | --- |
| ***Alcohols*** | |  |  |  |  |  |  |
| Isopropyl Alcohol | | 0.00^d^ | 23.89^b^ | 9.06^c^ | 7.03^c^ | 60.02^a^ | 0.07342 |
| 1-Propanol | | 9.90 | 10.42 | 48.40 | 10.23 | 21.04 | 0.02754 |
| 1-Propanol, 2-methyl- | | 0.00^b^ | 0.00^b^ | 100.00^a^ | 0.00^b^ | 0.00^b^ | 0.00551 |
| 3-Buten-1-ol, 3-methyl- | | 0.00^b^ | 0.00^b^ | 100.00^a^ | 0.00^b^ | 0.00^b^ | 0.00024 |
| 1-Pentanol | | 59.14^a^ | 4.32^c^ | 15.31^b^ | 11.23^b^ | 10.00^b^ | <0.00001 |
| 4-Terpineol | | 0.00^c^ | 72.11^a^ | 0.00^c^ | 27.81^b^ | 0.00^c^ | 0.00037 |
| 2-Hexen-1-ol, (Z)- | | 57.79^a^ | 30.11^b^ | 0.00^d^ | 12.10^c^ | 0.00^d^ | 0.00005 |
| 2-Hexanol, 3-methyl- | | 32.00^ab^ | 48.00^a^ | 0.00^c^ | 20.00^b^ | 0.00^c^ | 0.00002 |
| 1-Hexanol | | 55.73^a^ | 14.29^b^ | 20.01^b^ | 7.46^c^ | 2.51^d^ | 0.00003 |
| Beta-linalool | | 0.00^c^ | 64.52^a^ | 0.00^c^ | 35.48^b^ | 0.00^c^ | 0.00018 |
| Cuminol | | 0.00^c^ | 72.55^a^ | 0.00^c^ | 27.45^b^ | 0.00^c^ | 0.00155 |
| 3-Heptanol | | 12.95^c^ | 53.83^a^ | 3.11^d^ | 30.11^b^ | 0.00^e^ | 0.02945 |
| Borneol | | 0.00^c^ | 69.00^a^ | 0.00^c^ | 31.00^b^ | 0.00^c^ | 0.01078 |
| 1-Nonanol | | 0.00^c^ | 10.15^b^ | 30.91^a^ | 14.02^b^ | 44.92^a^ | 0.00427 |
| 2-Nonen-1-ol, (E)- | | 0.00^b^ | 0.00^b^ | 100.00^a^ | 0.00^b^ | 0.00^b^ | 0.00037 |
| 1-Dodecanol | | 0.00^b^ | 0.00^b^ | 100.00^a^ | 0.00^b^ | 0.00^b^ | <0.00001 |
| Eucalyptol | | 0.00^c^ | 52.88^a^ | 0.00^c^ | 47.12^b^ | 0.00^c^ | 0.00569 |
| 1,8-Menthadien-4-ol | | 0.00^c^ | 62.47^a^ | 0.00^c^ | 37.53^b^ | 0.00^c^ | 0.01391 |
| ***Aldehydes*** | |  |  |  |  |  |  |
| Butanal | | 0.00^b^ | 0.00^b^ | 0.00^b^ | 0.00^b^ | 100.00^a^ | 0.00864 |
| 2-Butenal, 3-methyl- | | 62.05^a^ | 11.18^b^ | 0.00^c^ | 8.13^b^ | 18.65^b^ | 0.00698 |
| 2-Butenal, (Z)- | | 0.00^b^ | 0.00^b^ | 100.00^a^ | 0.00^b^ | 0.00^b^ | 0.00968 |
| 2-Butenal, 2-methyl- | | 68.14 | 10.10^bc^ | 0.00^c^ | 5.17^b^ | 16.59^b^ | <0.00001 |
| Pentanal | | 80.23^a^ | 5.86^b^ | 7.50^b^ | 2.13^b^ | 4.28^b^ | 0.00004 |
| Hexanal | | 56.00^a^ | 8.21^bc^ | 0.55^c^ | 1.10^b^ | 34.14^a^ | 0.00098 |
| 2-Hexenal | | 0.00^b^ | 0.00^b^ | 100.00^a^ | 0.00^b^ | 0.00^b^ | 0.00036 |
| Octanal | | 56.44^a^ | 0.00^b^ | 0.00^b^ | 0.00^b^ | 43.56^a^ | 0.00412 |
| 2-Octenal, (E)- | | 0.00^c^ | 61.12^a^ | 0.00^c^ | 38.88^b^ | 0.00^c^ | 0.00012 |
| Benzeneacetaldehyde | | 56.92^a^ | 0.00^b^ | 0.00^b^ | 0.00^b^ | 43.08^a^ | <0.00001 |
| Benzaldehyde, 3,4-dimethyl- | | 60.93^a^ | 12.44^b^ | 7.49^bc^ | 5.14^c^ | 14.00^b^ | 0.00049 |
| ***Ketones*** | |  |  |  |  |  |  |
| 2,3-Butanedione | | 10.81^bc^ | 11.12^abc^ | 68.93^a^ | 6.12^b^ | 3.02^c^ | 0.03688 |
| 2-Pentanone | | 42.65^a^ | 24.40^b^ | 18.54^b^ | 14.40^b^ | 0.00^c^ | 0.00218 |
| 2-Hexanone | | 0.86^c^ | 4.08^b^ | 94.70^a^ | 0.00^c^ | 0.35^c^ | <0.00001 |
| 2-Hexanone, 5-methyl- | | 78.16^a^ | 0.00^c^ | 0.00^c^ | 0.00^c^ | 21.84^b^ | <0.00001 |
| 2-Heptanone | | 29.54^b^ | 40.13^a^ | 1.11^c^ | 25.00^b^ | 4.23^c^ | 0.00001 |
| 2-Octanone | | 0.00^b^ | 0.00^b^ | 100.00^a^ | 0.00^b^ | 0.00^b^ | 0.00549 |
| 2,4-Heptanedione, 6-methyl- | | 0.00^b^ | 0.00^b^ | 100.00^a^ | 0.00^b^ | 0.00^b^ | 0.01297 |
| p-Menthone | | 0.00^c^ | 77.00^a^ | 0.00^c^ | 23.00^b^ | 0.00^c^ | 0.04972 |
| 2-Undecanone | | 0.00^c^ | 67.84^a^ | 0.00^c^ | 32.16^b^ | 0.00^c^ | 0.02230 |
| Acetophenone | | 0.00^c^ | 81.00^a^ | 0.00^c^ | 29.00^b^ | 0.00^c^ | 0.00115 |
|  | ***Others (alkanes, alkenes, sulfurates, amines, …)*** | | | | | | |
| 1-Decene | | 0.00^c^ | 60.40^a^ | 0.00^c^ | 39.60^b^ | 0.00^c^ | 0.00185 |
| 3-Dodecene, (E)- | | 0.00^c^ | 70.96^a^ | 0.00^c^ | 29.04^b^ | 0.00^c^ | 0.00391 |
| 7-Tetradecene, (Z)- | | 0.00^c^ | 80.00^a^ | 0.00^c^ | 20.00^b^ | 0.00^c^ | 0.00089 |
| D-Limonene | | 0.00^c^ | 69.77^a^ | 0.00^c^ | 20.23^b^ | 0.00^c^ | <0.00001 |
| Caryophillene | | 0.00^c^ | 82.10^a^ | 0.00^c^ | 17.90^b^ | 0.00^c^ | 0.00008 |
| Methanethiol | | 22.80^b^ | 56.20^a^ | 0.00^c^ | 21.00^b^ | 0.00^c^ | 0.02011 |
| Ethyl Acetate | | 68.04^a^ | 0.00^c^ | 20.36^b^ | 0.00^c^ | 11.61^bc^ | 0.00001 |
| Aniline | | 70.63^a^ | 3.02^c^ | 4.80^c^ | 3.09^c^ | 18.46^b^ | 0.00143 |
| 2-Acetylthiazole | | 0.00^c^ | 6.61^b^ | 29.60^a^ | 10.01^b^ | 53.77^a^ | 0.00907 |
| Butanamide, 3,3-dimethyl- | | 52.99^a^ | 0.00^b^ | 40.11^a^ | 6.90^b^ | 0.00^b^ | 0.00029 |
| Pyrazine | | 0.00^c^ | 9.23^b^ | 10.50^b^ | 7.25^b^ | 73.01^a^ | 0.00020 |
| Pyrazine, methyl- | | 0.00^d^ | 8.10^c^ | 25.30^b^ | 16.00^bc^ | 50.60^a^ | 0.02990 |
| Pyridine, 2,4,6-trimethyl- | | 0.00^b^ | 0.00^b^ | 0.00^b^ | 0.00^b^ | 100.00^a^ | <0.00001 |
| Heneicosane | | 28.73^a^ | 51.16^a^ | 0.00^c^ | 20.11^b^ | 0.00^c^ | <0.00001 |
| Tetracosane | | 0.00^c^ | 77.17^a^ | 0.78^c^ | 20.30^b^ | 1.75^c^ | 0.00050 |
| Eicosane | | 0.00^b^ | 55.00^a^ | 0.00^b^ | 45.00^a^ | 0.00^b^ | 0.00002 |

**p* value indicates MANOVA significance. ^abcd^ Different letters indicate statistical significance by Tukey’s HSD test (*p* < 0.05). HB = Hempseed bran; HBPA = HB protein extract hydrolyzed by alcalase; FOS = fructooligosaccharides; BC = Blank control.

**Table S6**. MANOVA categorical descriptors for the volatilome. categorized for the time of fermentation. % of contribution of VOCs descriptors significant among the time points.

| **VOC** | **0 h** | **6 h** | **18 h** | **24 h** | ***p* value*** |
| --- | --- | --- | --- | --- | --- |
| ***Alcohols*** |  |  |  |  |  |
| 1-Propanol, 3-(methylthio)- | 0.00^c^ | 14.19^b^ | 18.65^b^ | 67.16^a^ | 0.04120 |
| 1-Butanol, 3-methyl- | 0.00^cd^ | 6.64^c^ | 28.59^b^ | 64.77^a^ | 0.00222 |
| 1-Pentanol | 70.37^a^ | 9.42^b^ | 8.90^b^ | 11.30^b^ | <0.00001 |
| 2-Hexen-1-ol, (Z)- | 57.79^a^ | 2.97^c^ | 20.67^b^ | 18.57^b^ | 0.09274 |
| 1-Hexanol | 55.73^a^ | 18.48^b^ | 12.24^b^ | 13.54^b^ | 0.00628 |
| 1-Nonanol | 0.00^b^ | 32.78^a^ | 36.83^a^ | 30.39^a^ | 0.03760 |
| 1-Dodecanol | 0.00^c^ | 11.79^bc^ | 54.73^a^ | 33.48^ab^ | 0.04820 |
| Eucalyptol | 0.00^c^ | 8.24^bc^ | 25.72^b^ | 66.04 ^a^ | 0.04132 |
| 1,8-Menthadien-4-ol | 0.00^c^ | 11.78^b^ | 14.07^b^ | 74.15^a^ | 0.04173 |
| 1-Propanol, 3-(methylthio)- | 0.00^c^ | 14.19^b^ | 18.65^b^ | 67.16^a^ | 0.04120 |
| 1-Butanol, 3-methyl- | 0.00^c^ | 6.64^c^ | 28.59^b^ | 64.77^a^ | 0.00222 |
| ***Aldehydes*** |  |  |  |  |  |
| 2-Butenal, 3-methyl- | 62.05^a^ | 24.15^b^ | 1.68^c^ | 12.13^c^ | 0.00626 |
| 2-Butenal, 2-methyl- | 68.14^a^ | 15.05^b^ | 13.11^bc^ | 3.70^c^ | 0.03809 |
| Pentanal | 82.36^a^ | 17.64^b^ | 0.00^c^ | 0.00^c^ | <0.00001 |
| Hexanal | 56.00^a^ | 25.44^b^ | 17.68^b^ | 0.87^c^ | 0.01621 |
| Heptanal | 0.00^b^ | 92.92^a^ | 5.97^b^ | 1.11^b^ | 0.00008 |
| Nonanal | 22.39^ab^ | 53.78^a^ | 19.35^b^ | 4.47^b^ | 0.00254 |
| Benzaldehyde | 40.29^a^ | 38.00^a^ | 7.75^b^ | 13.97^ab^ | 0.01244 |
| ***Ketones*** |  |  |  |  |  |
| 3-Penten-2-one, 4-methyl- | 41.56^a^ | 14.62^b^ | 21.60^ab^ | 22.22^ab^ | 0.02732 |
| 2-Hexanone | 0.00^b^ | 14.70^b^ | 70.08^a^ | 15.22^b^ | 0.02009 |
| 2-Hexanone, 5-methyl- | 78.16^a^ | 5.23^b^ | 8.05^b^ | 8.56^b^ | 0.00013 |
| 4-Decanone | 0.00^b^ | 0.00^b^ | 21.75^ab^ | 78.25^a^ | 0.03151 |
| 2.4-Heptanedione, 6-methyl- | 0.00^b^ | 0.00^b^ | 62.75^a^ | 37.25^ab^ | 0.04127 |
| p-Menthone | 0.00^b^ | 16.15^b^ | 6.81^b^ | 77.04^a^ | 0.04913 |
| Acetophenone | 0.00^b^ | 2.96^b^ | 45.21^a^ | 51.83^a^ | 0.04298 |
| ***Others*** |  |  |  |  |  |
| Ethyl Acetate | 68.04^a^ | 3.10^b^ | 11.93^b^ | 16.93^b^ | 0.00010 |
| 2-Acetylthiazole | 0.00^b^ | 52.54^a^ | 28.34^ab^ | 19.12^ab^ | 0.10464 |
| Aniline | 73.63^a^ | 13.05^b^ | 9.43^b^ | 3.89^b^ | 0.00321 |
| Eicosane | 0.00^b^ | 26.72^ab^ | 23.97^ab^ | 49.31^a^ | 0.04948 |

**p* value indicates MANOVA significance; ^abcd^ Different letters indicate statistical significance by Tukey’s HSD test (*p* < 0.05).

**Table S7.** Significance of Spearman rank correlations

|  | Indole | Phenol | Phenol,  4-methyl- | Benzaldehyde | Benzene-  acetaldehyde | Phenol, 2,4-bis  (1,1-dimethyl  ethyl) | Acetic  acid | Propanoic  acid | Butanoic  acid | Pentanoic  acid | Hexanoic  acid | Butanoic acid,  2-methyl- | Pentanoic acid,  4-methyl- |
| --- | --- | --- | --- | --- | --- | --- | --- | --- | --- | --- | --- | --- | --- |
| *Bifidobacterium;s__*  *adolescentis* | -0.350000 | 0.142261 | -0.450000 | -0.493728 | 0.103510 | -0.416667 | 0.500298 | 0.457693 | 0.457693 | 0.474342 | 0.431291 | 0.000000 | 0.000000 |
| *Bifidobacterium;s__*  *bifidum* | 0.550000 | -0.058578 | -0.300000 | 0.209207 | 0.724569 | 0.483333 | 0.966092 | 0.915386 | 0.915386 | 0.948683 | 0.897085 | 0.821584 | 0.821584 |
| *Bacteroides;s__*  *acidifaciens* | -0.350000 | 0.142261 | -0.450000 | -0.493728 | 0.103510 | -0.416667 | 0.500298 | 0.457693 | 0.457693 | 0.474342 | 0.431291 | 0.000000 | 0.000000 |
| *Bacteroides;s__*  *fragilis* | 0.550000 | -0.058578 | -0.300000 | 0.209207 | 0.724569 | 0.483333 | 0.966092 | 0.915386 | 0.915386 | 0.948683 | 0.897085 | 0.821584 | 0.821584 |
| *Bacteroides;s__*  *thetaiotaomicron* | 0.550000 | -0.058578 | -0.300000 | 0.209207 | 0.724569 | 0.483333 | 0.966092 | 0.915386 | 0.915386 | 0.948683 | 0.897085 | 0.821584 | 0.821584 |
| *Bacteroides;s__*  *uniformis* | -0.350000 | 0.142261 | -0.450000 | -0.493728 | 0.103510 | -0.416667 | 0.500298 | 0.457693 | 0.457693 | 0.474342 | 0.431291 | 0.000000 | 0.000000 |
| *Parabacteroides;s__*  *distasonis* | -0.350000 | 0.142261 | -0.450000 | -0.493728 | 0.103510 | -0.416667 | 0.500298 | 0.457693 | 0.457693 | 0.474342 | 0.431291 | 0.000000 | 0.000000 |
| *Enterococcus;s__*  *durans* | -0.350000 | 0.142261 | -0.450000 | -0.493728 | 0.103510 | -0.416667 | 0.500298 | 0.457693 | 0.457693 | 0.474342 | 0.431291 | 0.000000 | 0.000000 |
| *Enterococcus;s__*  *faecalis* | 1.000000 | -0.008368 | -0.150000 | 0.861932 | 0.724569 | 0.933333 | 0.500298 | 0.457693 | 0.457693 | 0.474342 | 0.431291 | 0.821584 | 0.821584 |
| *Lactobacillus;s__*  *mucosae* | -0.135613 | 0.110647 | -0.423790 | -0.314919 | 0.315838 | -0.271225 | 0.631676 | 0.586207 | 0.551724 | 0.589662 | 0.526397 | 0.185695 | 0.185695 |
| *Lactobacillus;s__*  *plantarum* | -0.389887 | 0.025534 | -0.322080 | -0.553237 | 0.105279 | -0.457693 | 0.508850 | 0.465517 | 0.465517 | 0.482451 | 0.438664 | 0.000000 | 0.000000 |
| *Blautia;s__*  *obeum* | -0.350000 | 0.443519 | -0.300000 | 0.108788 | -0.517549 | -0.416667 | -0.897085 | -0.915386 | -0.915386 | -0.948683 | -0.966092 | -0.821584 | -0.821584 |
| *Roseburia;s__*  *faecis* | 0.950000 | 0.108788 | -0.333333 | 0.761513 | 0.724569 | 0.816667 | 0.603807 | 0.593306 | 0.525499 | 0.579751 | 0.534801 | 0.821584 | 0.821584 |
| *Ruminococcus;s__*  *torques* | 0.550000 | 0.242680 | -0.150000 | 0.811723 | 0.103510 | 0.483333 | -0.431291 | -0.457693 | -0.457693 | -0.474342 | -0.500298 | 0.000000 | 0.000000 |
| *Peptostreptococcaceae;g__*  *Clostridium;s__* | 0.550000 | 0.242680 | -0.150000 | 0.811723 | 0.103510 | 0.483333 | -0.431291 | -0.457693 | -0.457693 | -0.474342 | -0.500298 | 0.000000 | 0.000000 |
| *Faecalibacterium;s__*  *prausnitzii* | 0.400000 | 0.041841 | -0.450000 | 0.108788 | 0.621059 | 0.266667 | 0.879834 | 0.830628 | 0.796725 | 0.843274 | 0.776324 | 0.639010 | 0.639010 |
| *Collinsella;s__*  *aerofaciens* | -0.350000 | 0.443519 | -0.300000 | 0.108788 | -0.517549 | -0.416667 | -0.897085 | -0.915386 | -0.915386 | -0.948683 | -0.966092 | -0.821584 | -0.821584 |
| *Eggerthella;s__*  *lenta* | 0.550000 | 0.242680 | -0.150000 | 0.811723 | 0.103510 | 0.483333 | -0.431291 | -0.457693 | -0.457693 | -0.474342 | -0.500298 | 0.000000 | 0.000000 |
| *Coprobacillus;s__*  *cateniformis* | -0.350000 | 0.142261 | -0.450000 | -0.493728 | 0.103510 | -0.416667 | 0.500298 | 0.457693 | 0.457693 | 0.474342 | 0.431291 | 0.000000 | 0.000000 |
| *Sutterella;s__* | 0.550000 | -0.058578 | -0.300000 | 0.209207 | 0.724569 | 0.483333 | 0.966092 | 0.915386 | 0.915386 | 0.948683 | 0.897085 | 0.821584 | 0.821584 |
| *Bilophila;s__*  *wadsworthia* | -0.350000 | 0.443519 | -0.300000 | 0.108788 | -0.517549 | -0.416667 | -0.897085 | -0.915386 | -0.915386 | -0.948683 | -0.966092 | -0.821584 | -0.821584 |
| *Citrobacter;s__*  *freundii* | 0.550000 | 0.242680 | -0.150000 | 0.811723 | 0.103510 | 0.483333 | -0.431291 | -0.457693 | -0.457693 | -0.474342 | -0.500298 | 0.000000 | 0.000000 |
| *Escherichia;s__*  *albertii* | 0.550000 | 0.242680 | -0.150000 | 0.811723 | 0.103510 | 0.483333 | -0.431291 | -0.457693 | -0.457693 | -0.474342 | -0.500298 | 0.000000 | 0.000000 |
| *Desulfovibrio;s__* | -0.389887 | 0.025534 | -0.322080 | -0.553237 | 0.105279 | -0.457693 | 0.508850 | 0.465517 | 0.465517 | 0.482451 | 0.438664 | 0.000000 | 0.000000 |
| *Akkermansia;s__*  *muciniphila* | -0.350000 | 0.142261 | -0.450000 | -0.493728 | 0.103510 | -0.416667 | 0.500298 | 0.457693 | 0.457693 | 0.474342 | 0.431291 | 0.000000 | 0.000000 |

**Table S7 (continuation).** Significance of Spearman rank correlations

|  | 4-Terpineol | Beta-linalool | Cuminol | Borneol | Eucalyptol | 2-Hexenal | 2-Octenal, (E)- | p-Menthone | Aceto  phenone | D-Limonene | Caryophillene | 2-Nonen  -1-ol.(E)- |
| --- | --- | --- | --- | --- | --- | --- | --- | --- | --- | --- | --- | --- |
| *Bifidobacterium;s__*  *adolescentis* | 0.039606 | -0.039606 | 0.039606 | -0.039606 | 0.039606 | 0.841625 | 0.039606 | 0.039606 | 0.039606 | -0.039606 | 0.039606 | 0.762414 |
| *Bifidobacterium;s__*  *bifidum* | 0.841625 | 0.762414 | 0.841625 | 0.762414 | 0.841625 | 0.039606 | 0.841625 | 0.841625 | 0.841625 | 0.762414 | 0.841625 | -0.039606 |
| *Bacteroides;s__*  *acidifaciens* | 0.039606 | -0.039606 | 0.039606 | -0.039606 | 0.039606 | 0.841625 | 0.039606 | 0.039606 | 0.039606 | -0.039606 | 0.039606 | 0.762414 |
| *Bacteroides;s__*  *fragilis* | 0.841625 | 0.762414 | 0.841625 | 0.762414 | 0.841625 | 0.039606 | 0.841625 | 0.841625 | 0.841625 | 0.762414 | 0.841625 | -0.039606 |
| *Bacteroides;s__*  *thetaiotaomicron* | 0.841625 | 0.762414 | 0.841625 | 0.762414 | 0.841625 | 0.039606 | 0.841625 | 0.841625 | 0.841625 | 0.762414 | 0.841625 | -0.039606 |
| *Bacteroides;s__*  *uniformis* | 0.039606 | -0.039606 | 0.039606 | -0.039606 | 0.039606 | 0.841625 | 0.039606 | 0.039606 | 0.039606 | -0.039606 | 0.039606 | 0.762414 |
| *Parabacteroides;s__*  *distasonis* | 0.039606 | -0.039606 | 0.039606 | -0.039606 | 0.039606 | 0.841625 | 0.039606 | 0.039606 | 0.039606 | -0.039606 | 0.039606 | 0.762414 |
| *Enterococcus;s__*  *durans* | 0.039606 | -0.039606 | 0.039606 | -0.039606 | 0.039606 | 0.841625 | 0.039606 | 0.039606 | 0.039606 | -0.039606 | 0.039606 | 0.762414 |
| *Enterococcus;s__*  *faecalis* | 0.841625 | 0.762414 | 0.841625 | 0.762414 | 0.841625 | -0.762414 | 0.841625 | 0.841625 | 0.841625 | 0.762414 | 0.841625 | -0.841625 |
| *Lactobacillus;s__*  *mucosae* | 0.241698 | 0.120849 | 0.241698 | 0.120849 | 0.241698 | 0.715023 | 0.241698 | 0.241698 | 0.241698 | 0.120849 | 0.241698 | 0.553891 |
| *Lactobacillus;s__*  *plantarum* | 0.040283 | -0.040283 | 0.040283 | -0.040283 | 0.040283 | 0.856013 | 0.040283 | 0.040283 | 0.040283 | -0.040283 | 0.040283 | 0.775447 |
| *Blautia;s__*  *obeum* | -0.762414 | -0.841625 | -0.762414 | -0.841625 | -0.762414 | 0.039606 | -0.762414 | -0.762414 | -0.762414 | -0.841625 | -0.762414 | -0.039606 |
| *Roseburia;s__*  *faecis* | 0.841625 | 0.762414 | 0.841625 | 0.762414 | 0.841625 | -0.544581 | 0.841625 | 0.841625 | 0.841625 | 0.762414 | 0.841625 | -0.703005 |
| *Ruminococcus;s__*  *torques* | 0.039606 | -0.039606 | 0.039606 | -0.039606 | 0.039606 | -0.762414 | 0.039606 | 0.039606 | 0.039606 | -0.039606 | 0.039606 | -0.841625 |
| *Peptostreptococcaceae;g__*  *Clostridium;s__* | 0.039606 | -0.039606 | 0.039606 | -0.039606 | 0.039606 | -0.762414 | 0.039606 | 0.039606 | 0.039606 | -0.039606 | 0.039606 | -0.841625 |
| *Faecalibacterium;s__*  *prausnitzii* | 0.683202 | 0.564384 | 0.683202 | 0.564384 | 0.683202 | 0.257438 | 0.683202 | 0.683202 | 0.683202 | 0.564384 | 0.683202 | 0.099015 |
| *Collinsella;s__*  *aerofaciens* | -0.762414 | -0.841625 | -0.762414 | -0.841625 | -0.762414 | 0.039606 | -0.762414 | -0.762414 | -0.762414 | -0.841625 | -0.762414 | -0.039606 |
| *Eggerthella;s__*  *lenta* | 0.039606 | -0.039606 | 0.039606 | -0.039606 | 0.039606 | -0.762414 | 0.039606 | 0.039606 | 0.039606 | -0.039606 | 0.039606 | -0.841625 |
| *Coprobacillus;s__*  *cateniformis* | 0.039606 | -0.039606 | 0.039606 | -0.039606 | 0.039606 | 0.841625 | 0.039606 | 0.039606 | 0.039606 | -0.039606 | 0.039606 | 0.762414 |
| *Sutterella;s__* | 0.841625 | 0.762414 | 0.841625 | 0.762414 | 0.841625 | 0.039606 | 0.841625 | 0.841625 | 0.841625 | 0.762414 | 0.841625 | -0.039606 |
| *Bilophila;s__*  *wadsworthia* | -0.762414 | -0.841625 | -0.762414 | -0.841625 | -0.762414 | 0.039606 | -0.762414 | -0.762414 | -0.762414 | -0.841625 | -0.762414 | -0.039606 |
| *Citrobacter;s__*  *freundii* | 0.039606 | -0.039606 | 0.039606 | -0.039606 | 0.039606 | -0.762414 | 0.039606 | 0.039606 | 0.039606 | -0.039606 | 0.039606 | -0.841625 |
| *Escherichia;s__*  *albertii* | 0.039606 | -0.039606 | 0.039606 | -0.039606 | 0.039606 | -0.762414 | 0.039606 | 0.039606 | 0.039606 | -0.039606 | 0.039606 | -0.841625 |
| *Desulfovibrio;s__* | 0.040283 | -0.040283 | 0.040283 | -0.040283 | 0.040283 | 0.856013 | 0.040283 | 0.040283 | 0.040283 | -0.040283 | 0.040283 | 0.775447 |
| *Akkermansia;s__*  *muciniphila* | 0.039606 | -0.039606 | 0.039606 | -0.039606 | 0.039606 | 0.841625 | 0.039606 | 0.039606 | 0.039606 | -0.039606 | 0.039606 | 0.762414 |

**Table S8.** Descriptive Statistics and Shapiro-Wilk’s W Test for Normality of Distribution of Volatile Organic Compounds

|  | Shapiro-Wilk’s | **Mean** | **Minimum** | **Maximum** | **Std.Dev.** |
| --- | --- | --- | --- | --- | --- |
| Ethyl alcohol | W=0.86648. p=0.01020 | 0.334957 | 0.013966 | 1.000000 | 0.312989 |
| Ethanol, 2-(methylthio)- | W=0.92876. p=0.14614 | 0.006132 | 0.000000 | 0.016914 | 0.004800 |
| Isopropyl alcohol | W=0.67618. p=0.00002 | 0.159978 | 0.000000 | 0.979357 | 0.236713 |
| 1-Propanol | W=0.73074. p=0.00009 | 0.074748 | 0.005701 | 0.344976 | 0.086099 |
| 1-Propanol, 2-methyl- | W=0.53844. p=0.00001 | 0.002493 | 0.000000 | 0.021524 | 0.005337 |
| 1-Propanol, 3-(methylthio)- | W=0.68510. p=0.00003 | 0.003521 | 0.000000 | 0.021863 | 0.005636 |
| 1-Butanol | W=0.91716. p=0.08737 | 0.232269 | 0.019485 | 0.585088 | 0.145365 |
| 1-Butanol. 3-methyl- | W=0.81495. p=0.00146 | 0.067579 | 0.000000 | 0.201544 | 0.074893 |
| 3-Buten-1-ol, 3-methyl- | W=0.60760. p=0.00000 | 0.002379 | 0.000000 | 0.014402 | 0.004484 |
| 1-Pentanol | W=0.67115. p=0.00002 | 0.066274 | 0.005525 | 0.332560 | 0.085168 |
| 4-Terpineol | W=0.59459. p=0.00001 | 0.003378 | 0.000000 | 0.020013 | 0.006455 |
| 2-Hexen-1-ol, (Z)- | W=0.66254. p=0.00001 | 0.013233 | 0.000000 | 0.047959 | 0.019565 |
| 2-Hexanol, 3-methyl- | W=0.72076. p=0.00007 | 0.005356 | 0.000000 | 0.025883 | 0.008074 |
| 1-Hexanol | W=0.86322. p=0.00895 | 0.163376 | 0.008682 | 0.599912 | 0.153620 |
| Beta-linalool | W=0.60698. p=0.00000 | 0.004295 | 0.000000 | 0.024859 | 0.008032 |
| Cuminol | W=0.56834. p=0.00000 | 0.003589 | 0.000000 | 0.022633 | 0.007223 |
| 1-Hexanol, 2-ethyl- | W=0.90592. p=0.05332 | 0.011144 | 0.003281 | 0.027159 | 0.005523 |
| 3-Heptanol | W=0.53211. p=0.00000 | 0.003179 | 0.000000 | 0.028471 | 0.006821 |
| 1-Heptanol | W=0.82439. p=0.00204 | 0.131174 | 0.013198 | 0.428129 | 0.129449 |
| 1-Octanol | W=0.86836. p=0.01099 | 0.049886 | 0.012957 | 0.125414 | 0.035492 |
| Borneol | W=0.52193. p=0.00000 | 0.010793 | 0.000000 | 0.101934 | 0.024089 |
| 1-Nonanol | W=0.91030. p=0.06457 | 0.054097 | 0.000000 | 0.148891 | 0.046059 |
| 2-Nonen-1-ol, (E)- | W=0.60119. p=0.00000 | 0.003167 | 0.000000 | 0.023742 | 0.006053 |
| 1-Dodecanol | W=0.61780. p=0.00000 | 0.009760 | 0.000000 | 0.037401 | 0.015387 |
| Eucalyptol | W=0.52688. p=0.00000 | 0.007040 | 0.000000 | 0.047108 | 0.015100 |
| 1,8-Menthadien-4-ol | W=0.50643. p=0.00000 | 0.008331 | 0.000000 | 0.066466 | 0.018931 |
| Phenylethyl alcohol | W=0.95020. p=0.37018 | 0.028479 | 0.004475 | 0.060756 | 0.014462 |
| Butanal | W=0.53592. p=0.00000 | 0.002196 | 0.000000 | 0.019378 | 0.004830 |
| Butanal, 2-methyl- | W=0.58264. p=0.00000 | 0.002487 | 0.000000 | 0.019642 | 0.004566 |
| 2-Butenal, 3-methyl- | W=0.70557. p=0.00005 | 0.005026 | 0.000000 | 0.018971 | 0.007228 |
| 2-Butenal, (Z)- | W=0.62373. p=0.00001 | 0.000928 | 0.000000 | 0.006230 | 0.001719 |
| 2-Butenal, 2-methyl- | W=0.77787. p=0.00041 | 0.003485 | 0.000000 | 0.015343 | 0.004484 |
| Pentanal | W=0.56709. p=0.00000 | 0.015268 | 0.000000 | 0.127912 | 0.030600 |
| Hexanal | W=0.50866. p=0.00000 | 0.107320 | 0.000000 | 1.000000 | 0.246791 |
| 2-Hexenal | W=0.32545. p=0.00000 | 0.002797 | 0.000000 | 0.043273 | 0.009757 |
| Heptanal | W=0.70591. p=0.00005 | 0.012426 | 0.000000 | 0.073357 | 0.018981 |
| Octanal | W=0.62775. p=0.00001 | 0.004526 | 0.000000 | 0.021242 | 0.007736 |
| Nonanal | W=0.81246. p=0.00133 | 0.018872 | 0.001212 | 0.060737 | 0.019537 |
| 2-Octenal, (E)- | W=0.60630. p=0.00000 | 0.015247 | 0.000000 | 0.089529 | 0.028240 |
| Benzaldehyde | W=0.87373. p=0.01367 | 0.051589 | 0.002342 | 0.159374 | 0.047887 |
| Benzeneacetaldehyde | W=0.74418. p=0.00014 | 0.021427 | 0.000000 | 0.085074 | 0.029360 |
| Benzaldehyde, 4-methyl- | W=0.60546. p=0.00000 | 0.005560 | 0.000000 | 0.042676 | 0.010631 |
| Benzaldehyde, 3,4-dimethyl- | W=0.47954. p=0.00000 | 0.012564 | 0.000000 | 0.109542 | 0.023772 |
| Acetone | W=0.64432. p=0.00001 | 0.076370 | 0.010649 | 0.381722 | 0.101266 |
| 2-Butanone | W=0.75715. p=0.00021 | 0.034405 | 0.001374 | 0.156629 | 0.038963 |
| 2,3-Butanedione | W=0.63656. p=0.00001 | 0.017353 | 0.000000 | 0.116192 | 0.027669 |
| 2-Pentanone | W=0.87383. p=0.01373 | 0.018129 | 0.000000 | 0.059851 | 0.018755 |
| 2-Hexanone | W=0.67470. p=0.00002 | 0.208909 | 0.000000 | 1.000000 | 0.331533 |
| 3-Penten-2-one, 4-methyl- | W=0.91266. p=0.07164 | 0.004604 | 0.000000 | 0.012125 | 0.003507 |
| 2-Hexanone, 4-methyl- | W=0.42519. p=0.00000 | 0.013035 | 0.000000 | 0.108948 | 0.032841 |
| 2-Hexanone, 5-methyl- | W=0.61920. p=0.00000 | 0.014388 | 0.000000 | 0.107115 | 0.025960 |
| 2-Heptanone | W=0.75771. p=0.00022 | 0.007163 | 0.000000 | 0.027725 | 0.009628 |
| 2,3-Hexanedione | W=0.42840. p=0.00000 | 0.001759 | 0.000000 | 0.020680 | 0.004699 |
| 2-Octanone | W=0.52539. p=0.00000 | 0.003213 | 0.000000 | 0.020632 | 0.006878 |
| 2.4-Heptanedione, 6-methyl- | W=0.52068. p=0.00000 | 0.002375 | 0.000000 | 0.020014 | 0.005370 |
| *p*-Menthone | W=0.37723. p=0.00000 | 0.002520 | 0.000000 | 0.033577 | 0.007516 |
| 4-Decanone | W=0.46752. p=0.00000 | 0.001974 | 0.000000 | 0.020262 | 0.005042 |
| 2-Undecanone | W=0.47804. p=0.00000 | 0.001203 | 0.000000 | 0.012389 | 0.002836 |
| Acetophenone | W=0.53450. p=0.00000 | 0.009621 | 0.000000 | 0.050280 | 0.019133 |
| 1-Decene | W=0.57794. p=0.00000 | 0.014121 | 0.000000 | 0.106886 | 0.028636 |
| 3-Dodecene, (E)- | W=0.55818. p=0.00000 | 0.009435 | 0.000000 | 0.071257 | 0.019827 |
| 7-Tetradecene, (Z)- | W=0.57182. p=0.00000 | 0.006009 | 0.000000 | 0.035558 | 0.011832 |
| D-Limonene | W=0.60277. p=0.00000 | 0.017596 | 0.000000 | 0.076214 | 0.030357 |
| Caryophillene | W=0.62316. p=0.0000 | 0.001822 | 0.000000 | 0.012525 | 0.003342 |
| Methanethiol | W=0.54954. p=0.00000 | 0.007042 | 0.000000 | 0.047796 | 0.014630 |
| Disulfide dimethyl- | W=0.64592. p=0.00001 | 0.226744 | 0.000000 | 1.000000 | 0.370628 |
| Dimethyl sulfide | W=0.71855. p=0.00007 | 0.002603 | 0.000000 | 0.010989 | 0.003828 |
| Dimethyl trisulfide | W=0.68357. p=0.00002 | 0.068120 | 0.000000 | 0.347588 | 0.105742 |
| Ethyl acetate | W=0.77116. p=0.00033 | 0.027066 | 0.000000 | 0.128656 | 0.036029 |
| 2-Acetylthiazole | W=0.83715. p=0.00327 | 0.050995 | 0.000000 | 0.164947 | 0.052169 |
| 1,2,4-Triazol-4-amine, N* | W=0.80436. p=0.00100 | 0.049457 | 0.000000 | 0.162561 | 0.058219 |
| Benzeneamine, N-ethyl- | W=0.77085. p=0.00033 | 0.015854 | 0.000000 | 0.076344 | 0.021555 |
| Aniline | W=0.56670. p=0.00000 | 0.012437 | 0.000000 | 0.098271 | 0.021935 |
| Butanamide, 3,3-dimethyl- | W=0.69728. p=0.00004 | 0.002696 | 0.000000 | 0.011618 | 0.004112 |
| Pyrazine | W=0.76957. p=0.00031 | 0.006061 | 0.000000 | 0.027308 | 0.008199 |
| Pyrazine, methyl- | W=0.83622. p=0.00316 | 0.006465 | 0.000000 | 0.023855 | 0.007278 |
| Pyridine, 2,4,6-trimethyl- | W=0.61387. p=0.00000 | 0.001860 | 0.000000 | 0.008054 | 0.003109 |
| Tetradecane | W=0.94736. p=0.32873 | 0.014573 | 0.001112 | 0.031219 | 0.006555 |
| Heneicosane | W=0.72484. p=0.00008 | 0.005916 | 0.000000 | 0.020842 | 0.007672 |
| Tetracosane | W=0.60431. p=0.00000 | 0.040945 | 0.000000 | 0.252754 | 0.076162 |
| Eicosane | W=0.64573. p=0.00001 | 0.010215 | 0.000000 | 0.047907 | 0.016851 |
| Indole | W=0.87527. p=0.00668 | 0.040100 | -1.000000 | 1.000000 | 0.604850 |
| Phenol | W=0.72660. p=0.00002 | -0.46900 | -1.000000 | 1.000000 | 0.680710 |
| *p*-Cresol | W=0.91499. p=0.04530 | -0.14810 | -1.000000 | 1.000000 | 0.543700 |
| Benzaldehyde | W=0.21431. p=0.00000 | -16.9171 | -398.0000 | 1.000000 | 81.17220 |
| 2,4-(DTBP) | W=0.94773. p=0.24178 | -0.17710 | -1.000000 | 1.000000 | 0.504060 |
| Acetic acid | W=0.82065. p=0.00065 | 0.250483 | 0.000000 | 1.000000 | 0.296921 |
| Propanoic acid | W=0.57984. p=0.00000 | 0.109377 | -0.046577 | 0.953423 | 0.250596 |
| Butanoic acid | W=0.56425. p=0.00000 | 0.084854 | -0.062301 | 0.937699 | 0.228698 |
| Pentanoic acid | W=0.70903. p=0.00001 | 0.247792 | 0.000000 | 1.000000 | 0.360193 |
| Hexanoic acid | W=0.64801. p=0.00000 | 0.163124 | 0.000000 | 1.000000 | 0.283350 |

*1,2,4-Triazol-4-amine, N-(2-thienylmethyl)-

**Table S9.** Levene's Test for Homogeneity of Variances of Volatile Organic Compounds

|  | **MS - Effect** | **MS - Error** | **F** | **p** |
| --- | --- | --- | --- | --- |
| Ethyl alcohol | 0.081509 | 0.011129 | 7.32399 | 0.002622 |
| Ethanol, 2-(methylthio)- | 0.000009 | 0.000003 | 2.86161 | 0.069566 |
| Isopropyl alcohol | 0.041333 | 0.020071 | 2.05935 | 0.146062 |
| 1-Propanol | 0.008360 | 0.001737 | 4.81309 | 0.014184 |
| 1-Propanol, 2-methyl- | 0.000027 | 0.000008 | 3.22359 | 0.050662 |
| 1-Propanol, 3-(methylthio)- | 0.000046 | 0.000010 | 4.52612 | 0.017612 |
| 1-Butanol | 0.010395 | 0.002617 | 3.97178 | 0.027190 |
| 1-Butanol, 3-methyl- | 0.001871 | 0.000991 | 1.88871 | 0.172199 |
| 3-Buten-1-ol, 3-methyl- | 0.000020 | 0.000002 | 10.00554 | 0.000593 |
| 1-Pentanol | 0.001293 | 0.000090 | 14.42905 | 0.000082 |
| 4-Terpineol | 0.000044 | 0.000005 | 9.66719 | 0.000705 |
| 2-Hexen-1-ol, (Z)- | 0.000339 | 0.000014 | 23.98861 | 0.000004 |
| 2-Hexanol, 3-methyl- | 0.000044 | 0.000004 | 10.28886 | 0.000514 |
| 1-Hexanol | 0.006723 | 0.002408 | 2.79149 | 0.074065 |
| Beta-linalool | 0.000064 | 0.000006 | 11.26370 | 0.000321 |
| Cuminol | 0.000080 | 0.000003 | 25.90405 | 0.000002 |
| 1-Hexanol, 2-ethyl- | 0.000002 | 0.000009 | 0.16627 | 0.917546 |
| 3-Heptanol | 0.000074 | 0.000010 | 7.19529 | 0.002837 |
| 1-Heptanol | 0.012759 | 0.003180 | 4.01272 | 0.026312 |
| 1-Octanol | 0.000654 | 0.000163 | 4.02356 | 0.026085 |
| Borneol | 0.000705 | 0.000160 | 4.39873 | 0.019423 |
| 1-Nonanol | 0.001597 | 0.000223 | 7.15125 | 0.002915 |
| 2-Nonen-1-ol, (E)- | 0.000027 | 0.000007 | 3.85658 | 0.029841 |
| 1-Dodecanol | 0.000009 | 0.000001 | 9.91384 | 0.000621 |
| Eucalyptol | 0.000428 | 0.000012 | 36.92521 | 0.000000 |
| 1,8-Menthadien-4-ol | 0.000720 | 0.000030 | 23.68946 | 0.000004 |
| Phenylethyl alcohol | 0.000226 | 0.000037 | 6.11082 | 0.005681 |
| Butanal | 0.000027 | 0.000006 | 4.14800 | 0.023633 |
| Butanal, 2-methyl- | 0.000035 | 0.000006 | 5.83900 | 0.006826 |
| 2-Butenal, 3-methyl- | 0.000037 | 0.000004 | 8.69800 | 0.001183 |
| 2-Butenal, (Z)- | 0.000002 | 0.000000 | 11.0590 | 0.000353 |
| 2-Butenal, 2-methyl- | 0.000005 | 0.000001 | 7.59300 | 0.002230 |
| Pentanal | 0.000432 | 0.000026 | 16.6540 | 0.000035 |
| Hexanal | 0.026435 | 0.000472 | 56.05300 | 0.000000 |
| 2-Hexenal | 0.000169 | 0.000000 | 6508.050 | 0.000000 |
| Heptanal | 0.000176 | 0.000127 | 1.38600 | 0.283047 |
| Octanal | 0.000082 | 0.000007 | 12.08600 | 0.000220 |
| Nonanal | 0.000310 | 0.000064 | 4.82100 | 0.014101 |
| 2-Octenal, (E)- | 0.000871 | 0.000036 | 24.37100 | 0.000003 |
| Benzaldehyde | 0.001480 | 0.000330 | 4.48200 | 0.018211 |
| Benzeneacetaldehyde | 0.000400 | 0.000016 | 25.10500 | 0.000003 |
| Benzaldehyde, 4-methyl- | 0.000227 | 0.000027 | 8.51100 | 0.001313 |
| Benzaldehyde, 3,4-dimethyl- | 0.000936 | 0.000005 | 195.805 | 0.000000 |
| Acetone | 0.008830 | 0.003780 | 2.33620 | 0.112388 |
| 2-Butanone | 0.002264 | 0.000224 | 10.0939 | 0.000567 |
| 2,3-Butanedione | 0.000978 | 0.000197 | 4.96140 | 0.012710 |
| 2-Pentanone | 0.000235 | 0.000027 | 8.76020 | 0.001143 |
| 2-Hexanone | 0.056518 | 0.003957 | 14.2817 | 0.000087 |
| 3-Penten-2-one, 4-methyl- | 0.000008 | 0.000004 | 2.00530 | 0.153843 |
| 2-Hexanone, 4-methyl- | 0.002779 | 0.000098 | 28.4874 | 0.000001 |
| 2-Hexanone, 5-methyl- | 0.000476 | 0.000002 | 198.1475 | 0.000000 |
| 2-Heptanone | 0.000044 | 0.000005 | 9.10160 | 0.000950 |
| 2,3-Hexanedione | 0.000034 | 0.000008 | 4.24440 | 0.021903 |
| 2-Octanone | 0.000073 | 0.000007 | 11.1135 | 0.000344 |
| 2,4-Heptanedione, 6-methyl- | 0.000044 | 0.000006 | 7.44680 | 0.002434 |
| *p*-Menthone | 0.000099 | 0.000022 | 4.45030 | 0.018667 |
| 4-Decanone | 0.000049 | 0.000006 | 8.57430 | 0.001267 |
| 2-Undecanone | 0.000011 | 0.000002 | 4.53950 | 0.017433 |
| Acetophenone | 0.000531 | 0.000022 | 23.6974 | 0.000004 |
| 1-Decene | 0.001141 | 0.000087 | 13.18408 | 0.000136 |
| 3-Dodecene, (E)- | 0.000625 | 0.000040 | 15.71303 | 0.000050 |
| 7-Tetradecene, (Z)- | 0.000213 | 0.000004 | 53.90826 | 0.000000 |
| D-Limonene | 0.000475 | 0.000064 | 7.436050 | 0.002450 |
| Caryophillene | 0.000006 | 0.000002 | 3.517704 | 0.039461 |
| Methanethiol | 0.000476 | 0.000009 | 55.44546 | 0.000000 |
| Disulfide dimethyl- | 0.203484 | 0.020734 | 9.81409 | 0.000653 |
| Dimethyl sulfide | 0.000024 | 0.000002 | 11.48936 | 0.000289 |
| Dimethyl trisulfide | 0.014136 | 0.002945 | 4.799740 | 0.014326 |
| Ethyl acetate | 0.000434 | 0.000069 | 6.294250 | 0.005030 |
| 2-Acetylthiazole | 0.001721 | 0.000500 | 3.443470 | 0.042004 |
| 1,2,4-Triazol-4-amine, N-(2-thienylmethyl)- | 0.005137 | 0.000346 | 14.86701 | 0.000069 |
| Benzeneamine, N-ethyl- | 0.000420 | 0.000153 | 2.75085 | 0.076820 |
| Aniline | 0.000686 | 0.000014 | 50.18218 | 0.000000 |
| Butanamide, 3,3-dimethyl- | 0.000017 | 0.000001 | 14.12485 | 0.000092 |
| Pyrazine | 0.000027 | 0.000004 | 6.937860 | 0.003328 |
| Pyrazine, methyl- | 0.000033 | 0.000011 | 3.067990 | 0.057984 |
| Pyridine, 2,4,6-trimethyl- | 0.000003 | 0.000001 | 3.914470 | 0.028474 |
| Tetradecane | 0.000043 | 0.000014 | 3.091420 | 0.056810 |
| Heneicosane | 0.000013 | 0.000001 | 15.92658 | 0.000046 |
| Tetracosane | 0.007226 | 0.000298 | 24.22179 | 0.000003 |
| Eicosane | 0.000077 | 0.000012 | 6.238100 | 0.005220 |
| Indole | 0.050000 | 0.015000 | 3.020705 | 0.053795 |
| Phenol | 0.100000 | 0.029000 | 3.543643 | 0.033152 |
| *p*-Cresol | 0.330000 | 0.045000 | 7.400217 | 0.001595 |
| Benzaldehyde | 18200.17 | 2929.239 | 6.213277 | 0.003713 |
| 2,4-(DTBP) | 0.260000 | 0.032000 | 8.367775 | 0.000840 |
| Acetic acid | 0.067856 | 0.010822 | 6.270240 | 0.003560 |
| Propanoic acid | 0.167854 | 0.006181 | 27.15677 | 0.000000 |
| Butanoic acid | 0.135225 | 0.006720 | 20.12420 | 0.000003 |
| Pentanoic acid | 0.204035 | 0.018740 | 10.88742 | 0.000187 |
| Hexanoic acid | 0.172268 | 0.009769 | 17.63326 | 0.000008 |

**Table S10.** Descriptive Statistics and Shapiro-Wilk’s W Test for Normality of Distribution of Microbiota at the Species level.

|  | **Shapiro - Wilk’s** | **Mean** | **Minimum** | **Maximum** | **Std.Dev.** |
| --- | --- | --- | --- | --- | --- |
| *Archaea;Other* | W=0.95708. p=0.76052 | 0.00263 | 0.000000 | 0.00603 | 0.00252 |
| *Methanobrevibacter;s__smithii* | W=0.84217. p=0.20179 | 0.00141 | 0.000829 | 0.00253 | 0.00079 |
| *Methanosphaera;s__stadtmanae* | W=0.84217. p=0.20179 | 0.00189 | 0.001314 | 0.00301 | 0.00079 |
| *Bifidobacterium;s__adolescentis* | W=0.72758. p=0.02327 | 7.69888 | 4.413893 | 15.36132 | 5.13689 |
| *Bifidobacterium;s__bifidum* | W=0.79621. p=0.09560 | 2.04860 | 0.817094 | 3.30738 | 1.33575 |
| *Bifidobacterium;s__longum* | W=0.86082. p=0.02631 | 3.32668 | 2.671006 | 4.14319 | 0.73365 |
| *Bacteroides;s__acidifaciens* | W=0.89282. p=0.39625 | 0.18780 | 0.094755 | 0.30084 | 0.09940 |
| *Bacteroides;s__caccae* | W=0.93714. p=0.63699 | 1.16280 | 0.411980 | 1.96609 | 0.71872 |
| *Bacteroides;s__fragilis* | W=0.99528. p=0.98267 | 0.53227 | 0.065917 | 1.00151 | 0.40058 |
| *Bacteroides;s__massiliensis* | W=0.80532. p=0.11212 | 0.51593 | 0.005255 | 1.00753 | 0.53241 |
| *Bacteroides;s__thetaiotaomicron* | W=0.84469. p=0.20943 | 2.97905 | 0.417715 | 7.87982 | 3.45826 |
| *Bacteroides;s__uniformis* | W=0.87595. p=0.32162 | 10.16720 | 0.499870 | 27.02591 | 11.82266 |
| *Parabacteroides;s__distasonis* | W=0.86386. p=0.27429 | 2.58539 | 0.932114 | 5.14444 | 2.04532 |
| *Enterococcus;s__durans* | W=0.86900. p=0.29378 | 3.24878 | 0.010326 | 8.20623 | 3.96163 |
| *Enterococcus;s__faecalis* | W=0.64063. p=0.00185 | 0.15284 | 0.000939 | 0.59818 | 0.29692 |
| *Lactobacillus;s__casei* | W=0.64309. p=0.00202 | 0.01297 | 0.000000 | 0.05094 | 0.02532 |
| *Lactobacillus;s__manihotivorans* | W=0.99143. p=0.96467 | 0.05986 | 0.051314 | 0.06959 | 0.00777 |
| *Lactobacillus;s__mucosae* | W=0.74773. p=0.03677 | 0.02862 | 0.000000 | 0.05657 | 0.03197 |
| *Lactobacillus;s__plantarum* | W=0.74844. p=0.03734 | 0.07111 | 0.000000 | 0.24151 | 0.11522 |
| *Streptococcus;s__pseudopneum** | W=0.78557. p=0.07876 | 0.00757 | 0.001507 | 0.02065 | 0.00888 |
| *Streptococcus;s__thermophilus* | W=0.88628. p=0.36614 | 0.26253 | 0.017078 | 0.66647 | 0.28282 |
| *Anaerococcus;s__hydrogenalis* | W=0.63880. p=0.00173 | 0.56936 | 0.013142 | 2.20739 | 1.09208 |
| *Blautia;s__* | W=0.76858. p=0.05678 | 2.02346 | 0.087392 | 6.32204 | 2.90388 |
| *Blautia;s__obeum* | W=0.84809. p=0.22004 | 0.34686 | 0.033149 | 0.74156 | 0.35762 |
| *Roseburia;s__faecis* | W=0.65214. p=0.00276 | 0.02159 | 0.003941 | 0.07228 | 0.03380 |
| *Ruminococcus;s__gnavus* | W=0.90264. p=0.44427 | 0.95746 | 0.052736 | 2.40303 | 1.06896 |
| *Ruminococcus;s__torques* | W=0.64468. p=0.00213 | 0.33264 | 0.001314 | 1.29726 | 0.64319 |
| *Peptostreptococcaceae;g__Clostr*** | W=0.96847. p=0.83198 | 0.08396 | 0.006568 | 0.16617 | 0.07115 |
| *Tepidibacter;s__* | W=0.87925. p=0.33544 | 1.57767 | 0.419070 | 2.53505 | 1.03578 |
| *Anaerotruncus;s__colihominis* | W=0.75388. p=0.04196 | 0.02882 | 0.012018 | 0.06687 | 0.02561 |
| *Faecalibacterium;s__prausnitzii* | W=0.95147. p=0.72532 | 2.20672 | 0.185391 | 4.25992 | 1.66373 |
| *Oscillospira;s__* | W=0.73755. p=0.02933 | 3.67672 | 0.254857 | 11.90182 | 5.53186 |
| *Ruminococcus;s__* | W=0.70362. p=0.01278 | 1.53061 | 0.273249 | 4.57797 | 2.03876 |
| *Dialister;s__invisus* | W=0.63868. p=0.00172 | 0.87714 | 0.010547 | 3.42620 | 1.69946 |
| *Megasphaera;s__elsdenii* | W=0.97000. p=0.84150 | 3.26680 | 0.204918 | 6.42812 | 2.77117 |
| *Megasphaera;s__hominis* | W=0.90673. p=0.46525 | 0.00233 | 0.001314 | 0.00375 | 0.00114 |
| *Collinsella;s__aerofaciens* | W=0.78895. p=0.08383 | 1.95830 | 0.467093 | 3.47437 | 1.62557 |
| *Eggerthella;s__lenta* | W=0.69104. p=0.00909 | 0.03726 | 0.003941 | 0.06866 | 0.03324 |
| *Coprobacillus;s__cateniformis* | W=0.81392. p=0.12963 | 0.48076 | 0.006866 | 1.05096 | 0.54771 |
| *Sutterella;s__* | W=0.88212. p=0.34777 | 2.40224 | 0.864376 | 4.93605 | 1.77021 |
| *Bilophila;s__wadsworthia* | W=0.89270. p=0.39569 | 0.36796 | 0.018081 | 0.94755 | 0.40859 |
| *Citrobacter;s__freundii* | W=0.94661. p=0.69504 | 0.06857 | 0.001314 | 0.16342 | 0.06949 |
| *Escherichia;s__* | W=0.79306. p=0.09034 | 14.13447 | 0.507829 | 41.93352 | 18.84431 |
| *Escherichia;s__albertii* | W=0.69104. p=0.00909 | 0.21415 | 0.042301 | 0.66603 | 0.30201 |
| *Desulfovibrio;s__* | W=0.89755. p=0.41901 | 0.15508 | 0.000000 | 0.39506 | 0.16926 |
| *Prevotella;s__bivia* | W=0.75732. p=0.04512 | 0.00726 | 0.000000 | 0.02441 | 0.01164 |
| *Prevotella;s__disiens* | W=0.64338. p=0.00204 | 0.03527 | 0.000000 | 0.13849 | 0.06882 |
| *Akkermansia;s__muciniphila* | W=0.83301. p=0.17580 | 0.84454 | 0.032070 | 1.31923 | 0.56051 |

** Streptococcus;s__pseudopneumoniae; ** Peptostreptococcaceae;g__Clostridium;s__*

**Table S11.** Levene's Test for Homogeneity of Variances of Microbiota at the species level.

|  | | | | |
| --- | --- | --- | --- | --- |
|  | **MS - Effect** | **MS - Error** | **F** | **p** |
| *Archaea;Other* | 0.000000 | 0.000000 | 0.857143 | 0.548922 |
| *Methanobrevibacter;s__smithii* | 0.031903 | 0.038030 | 0.838873 | 0.555711 |
| *Methanosphaera;s__stadtmanae* | 0.000002 | 0.000003 | 0.697983 | 0.612635 |
| *Bifidobacterium;s__adolescentis* | 0.575566 | 0.681472 | 0.844591 | 0.553572 |
| *Bifidobacterium;s__bifidum* | 0.004958 | 0.009369 | 0.529188 | 0.692863 |
| *Bifidobacterium;s__longum* | 0.045706 | 0.090745 | 0.503683 | 0.706247 |
| *Bacteroides;s__acidifaciens* | 0.000013 | 0.000029 | 0.440699 | 0.740789 |
| *Bacteroides;s__caccae* | 0.001183 | 0.002132 | 0.554982 | 0.679675 |
| *Bacteroides;s__fragilis* | 0.006477 | 0.008552 | 0.757357 | 0.587620 |
| *Bacteroides;s__massiliensis* | 0.010499 | 0.014872 | 0.705968 | 0.609179 |
| *Bacteroides;s__thetaiotaomicron* | 0.001516 | 0.002254 | 0.672595 | 0.623816 |
| *Bacteroides;s__uniformis* | 0.017244 | 0.020135 | 0.856421 | 0.549188 |
| *Parabacteroides;s__distasonis* | 0.010736 | 0.023656 | 0.453854 | 0.733398 |
| *Enterococcus;s__durans* | 0.085384 | 0.099623 | 0.857072 | 0.548949 |
| *Enterococcus;s__faecalis* | 0.000008 | 0.000010 | 0.823287 | 0.561604 |
| *Lactobacillus;s__casei* | 0.000090 | 0.000106 | 0.847169 | 0.552613 |
| *Lactobacillus;s__manihotivorans* | 0.000185 | 0.000221 | 0.836138 | 0.556738 |
| *Lactobacillus;s__mucosae* | 0.000001 | 0.000001 | 0.745804 | 0.592366 |
| *Lactobacillus;s__plantarum* | 0.000000 | 0.000000 | 0.777747 | 0.579382 |
| *Streptococcus;s__pseudopneumoniae* | 0.000956 | 0.001342 | 0.712515 | 0.606367 |
| *Streptococcus;s__thermophilus* | 0.023661 | 0.030857 | 0.766784 | 0.583789 |
| *Anaerococcus;s__hydrogenalis* | 0.000003 | 0.000006 | 0.522266 | 0.696461 |
| *Blautia;s__* | 0.453472 | 0.945830 | 0.479443 | 0.719289 |
| *Blautia;s__obeum* | 0.007948 | 0.009336 | 0.851308 | 0.551077 |
| *Roseburia;s__faecis* | 0.000232 | 0.000281 | 0.825324 | 0.560829 |
| *Ruminococcus;s__gnavus* | 0.091222 | 0.132892 | 0.686438 | 0.617682 |
| *Ruminococcus;s__torques* | 0.053265 | 0.121412 | 0.438709 | 0.741915 |
| *Peptostreptococcaceae;g__Clostridium;s__* | 0.000421 | 0.000862 | 0.488021 | 0.714638 |
| *Tepidibacter;s__* | 0.032596 | 0.055696 | 0.585246 | 0.664641 |
| *Anaerotruncus;s__colihominis* | 0.000000 | 0.000000 | 0.834327 | 0.557420 |
| *Faecalibacterium;s__prausnitzii* | 0.100634 | 0.160622 | 0.626529 | 0.644881 |
| *Oscillospira;s__* | 0.002657 | 0.003185 | 0.834020 | 0.557536 |
| *Ruminococcus;s__* | 0.407435 | 0.622150 | 0.654883 | 0.631795 |
| *Dialister;s__invisus* | 0.049169 | 0.113452 | 0.433391 | 0.744935 |
| *Megasphaera;s__elsdenii* | 1.322003 | 1.543586 | 0.856449 | 0.549178 |
| *Megasphaera;s__hominis* | 0.000000 | 0.000000 | 0.624022 | 0.646057 |
| *Collinsella;s__aerofaciens* | 0.106183 | 0.124208 | 0.854885 | 0.549754 |
| *Eggerthella;s__lenta* | 0.000042 | 0.000051 | 0.822375 | 0.561952 |
| *Coprobacillus;s__cateniformis* | 0.000116 | 0.000210 | 0.554724 | 0.679805 |
| *Sutterella;s__* | 0.054746 | 0.108604 | 0.504082 | 0.706035 |
| *Bilophila;s__wadsworthia* | 0.000066 | 0.000085 | 0.772805 | 0.581362 |
| *Citrobacter;s__freundii* | 0.000168 | 0.000203 | 0.827916 | 0.559844 |
| *Escherichia;s__* | 0.001893 | 0.002386 | 0.793554 | 0.573116 |
| *Escherichia;s__albertii* | 0.000339 | 0.000411 | 0.824640 | 0.561089 |
| *Desulfovibrio;s__* | 0.001958 | 0.004568 | 0.428571 | 0.747684 |
| *Prevotella;s__bivia* | 0.000033 | 0.000038 | 0.851855 | 0.550874 |
| *Prevotella;s__disiens* | 0.001208 | 0.001409 | 0.857011 | 0.548971 |
| *Akkermansia;s__muciniphila* | 0.002353 | 0.004428 | 0.531356 | 0.691741 |


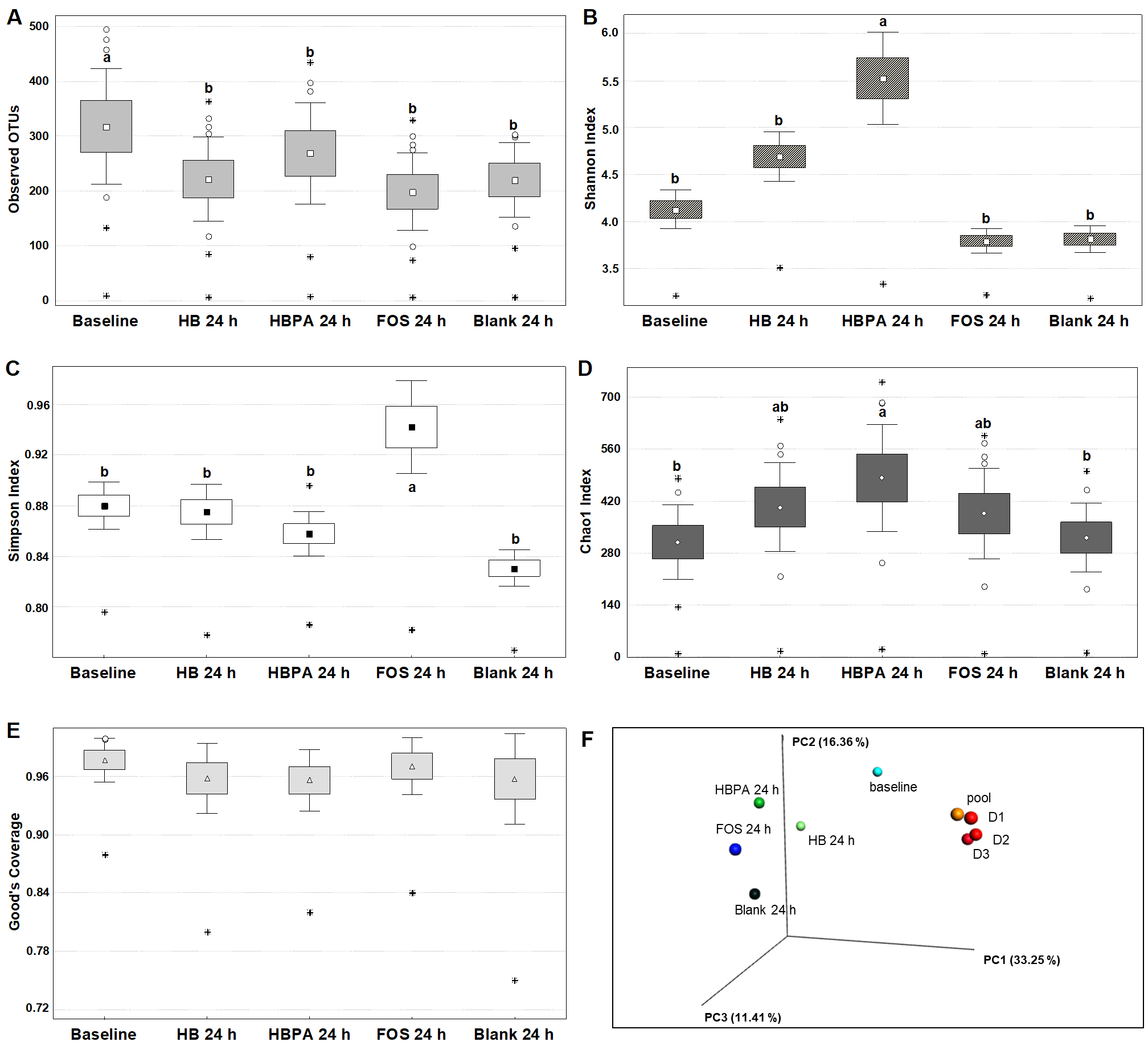


**Figure S1.** Microbiota alpha and beta diversities. A – E = alpha diversity indexes: F = beta diversity Bray Curtis PCoA. Box = mean ± Standard Error; whiskers = Confidence Interval (95%); dots = Outliers; asterisks = extremes. ^ab^ Different letters on the plots indicates statistical significance by Student’s t-test (*p* < 0.05). HB = Hempseed bran; HBPA = HB protein extract hydrolyzed by alcalase; FOS = fructooligosaccharides; Blank = Blank control.

**
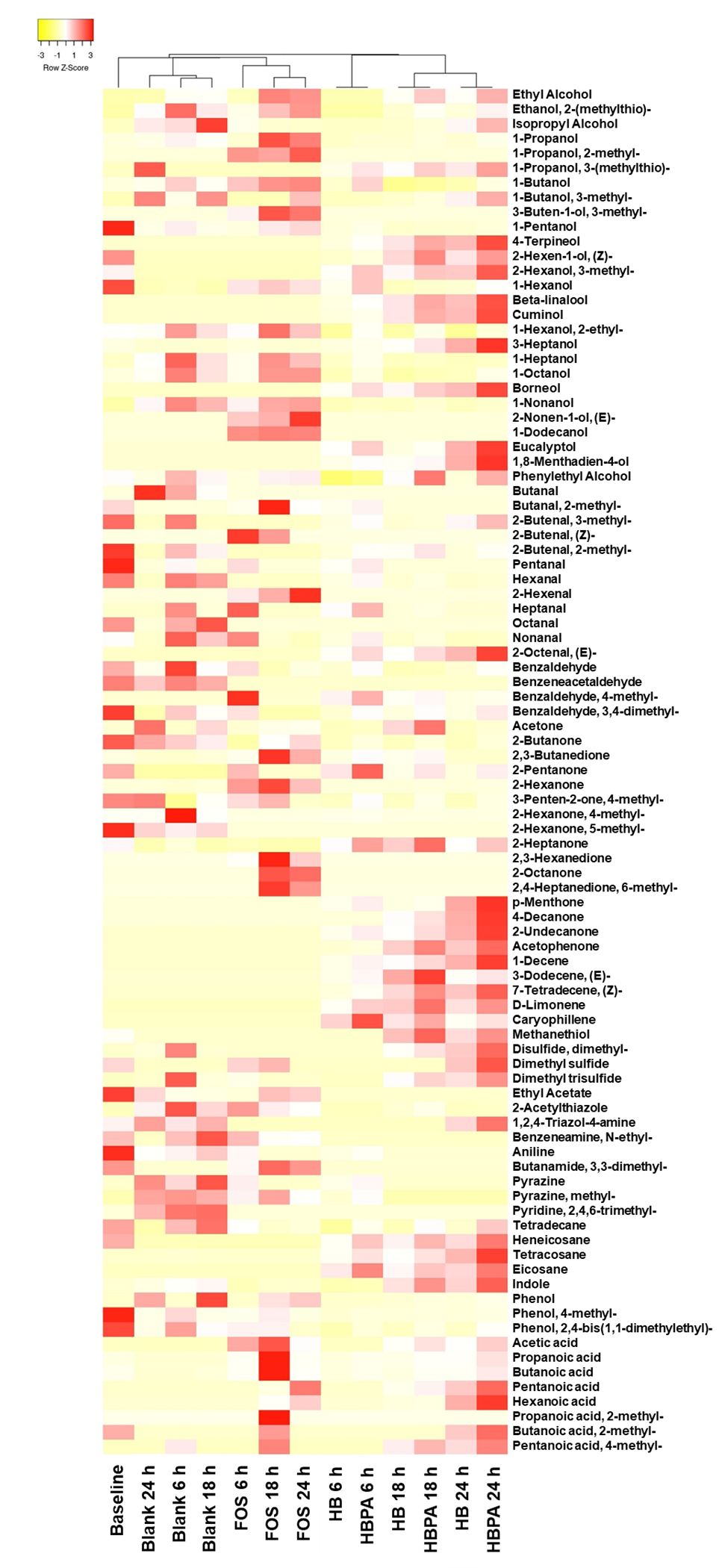
**

**Figure S2.** Quantification heatmap of total VOCs. HB = Hempseed bran; HBPA = HB protein extract hydrolyzed by alcalase; FOS = fructooligosaccharides; Blank = Blank control. Heat map was generated with the Expression tool on <http://heatmapper.ca/expression/> (last accessed on 2 January 2023).

**References**

1. Lane. D.J. *et al.* Evolutionary relationships among sulfur- and iron-oxidizing eubacteria. *J. Bacteriol.* **174**. 269–278. <https://doi.org/10.1128/jb.174.1.269-278.1992> (1992).
2. Bartosch. S. *et al.* Characterization of Bacterial Communities in Feces from Healthy Elderly Volunteers and Hospitalized Elderly Patients by Using Real-Time PCR and Effects of Antibiotic Treatment on the Fecal Microbiota. *Appl. Environ. Microbiol.* **70**. 3575–3581.
   <https://doi.org/10.1128/AEM.70.6.3575-3581.2004> (2004).
3. Walter. J. *et al.* Detection of *Lactobacillus. Pediococcus. Leuconostoc. and Weissella* Species in Human Feces by Using Group-Specific PCR Primers and Denaturing Gradient Gel Electrophoresis. *Appl. Environ. Microbiol.* **67**. 2578–2585. <https://doi.org/10.1128/AEM.67.6.2578-2585.2001> (2001).
4. Masco. L. *et al.* Polyphasic taxonomic analysis of Bifidobacterium animalis and Bifidobacterium lactis reveals relatedness at the subspecies level: Reclassification of *Bifidobacterium animalis as Bifidobacterium animalis subsp. animalis subsp. nov. and Bifidobacterium lactis as Bifidobacterium animalis subsp. lactis subsp. nov. Int. J. Syst. Evol. Microbiol.* **54**. 1137–1143. <https://doi.org/10.1099/ijs.0.03011-0> (2004).
